# Hetero-Multivalency of *Pseudomonas aeruginosa* Lectin LecA Binding to Model Membranes

**DOI:** 10.1038/s41598-018-26643-7

**Published:** 2018-05-30

**Authors:** Nolan C. Worstell, Akshi Singla, Panatda Saenkham, Thushara Galbadage, Preeti Sule, Dongheon Lee, Alec Mohr, Joseph Sang-Il Kwon, Jeffrey D. Cirillo, Hung-Jen Wu

**Affiliations:** 1 Department of Chemical Engineering, Texas A&M University, College Station, Texas USA; 2 Department of Microbial Pathogenesis and Immunology, Texas A&M Health Science Center, Bryan, Texas USA

## Abstract

A single glycan-lectin interaction is often weak and semi-specific. Multiple binding domains in a single lectin can bind with multiple glycan molecules simultaneously, making it difficult for the classic “lock-and-key” model to explain these interactions. We demonstrated that hetero-multivalency, a homo-oligomeric protein simultaneously binding to at least two types of ligands, influences LecA (a *Pseudomonas aeruginosa* adhesin)-glycolipid recognition. We also observed enhanced binding between *P. aeruginosa* and mixed glycolipid liposomes. Interestingly, strong ligands could activate weaker binding ligands leading to higher LecA binding capacity. This hetero-multivalency is probably mediated via a simple mechanism, Reduction of Dimensionality (RD). To understand the influence of RD, we also modeled LecA’s two-step binding process with membranes using a kinetic Monte Carlo simulation. The simulation identified the frequency of low-affinity ligand encounters with bound LecA and the bound LecA’s retention of the low-affinity ligand as essential parameters for triggering hetero-multivalent binding, agreeing with experimental observations. The hetero-multivalency can alter lectin binding properties, including avidities, capacities, and kinetics, and therefore, it likely occurs in various multivalent binding systems. Using hetero-multivalency concept, we also offered a new strategy to design high-affinity drug carriers for targeted drug delivery.

## Introduction

*Pseudomonas aeruginosa* is a ubiquitous and opportunistic bacterium. The increase of antibiotic resistance worldwide limits therapeutic options, leading to high morbidity and mortality of *P. aeruginosa* infections^1,2^. One mechanism that *P. aeruginosa* uses to cause disease is adhesion to epithelial cells^3–6^. Adhesion of *P. aeruginosa* is mediated by surface adhesins, including LecA (i.e. PA-IL), LecB (i.e. PA-IIL), and Type IV Pilus (T4P), which bind to glycan ligands on epithelial cell surfaces^7–11^. In addition to their role in adhesion, LecA and LecB can influence host cell functions^11–16^. Thus, it is essential for us to understand the binding mechanisms for *P. aeruginosa* adhesins to host cell ligands in order to gain insight into strategies to combat infections.

**Figure 1:**
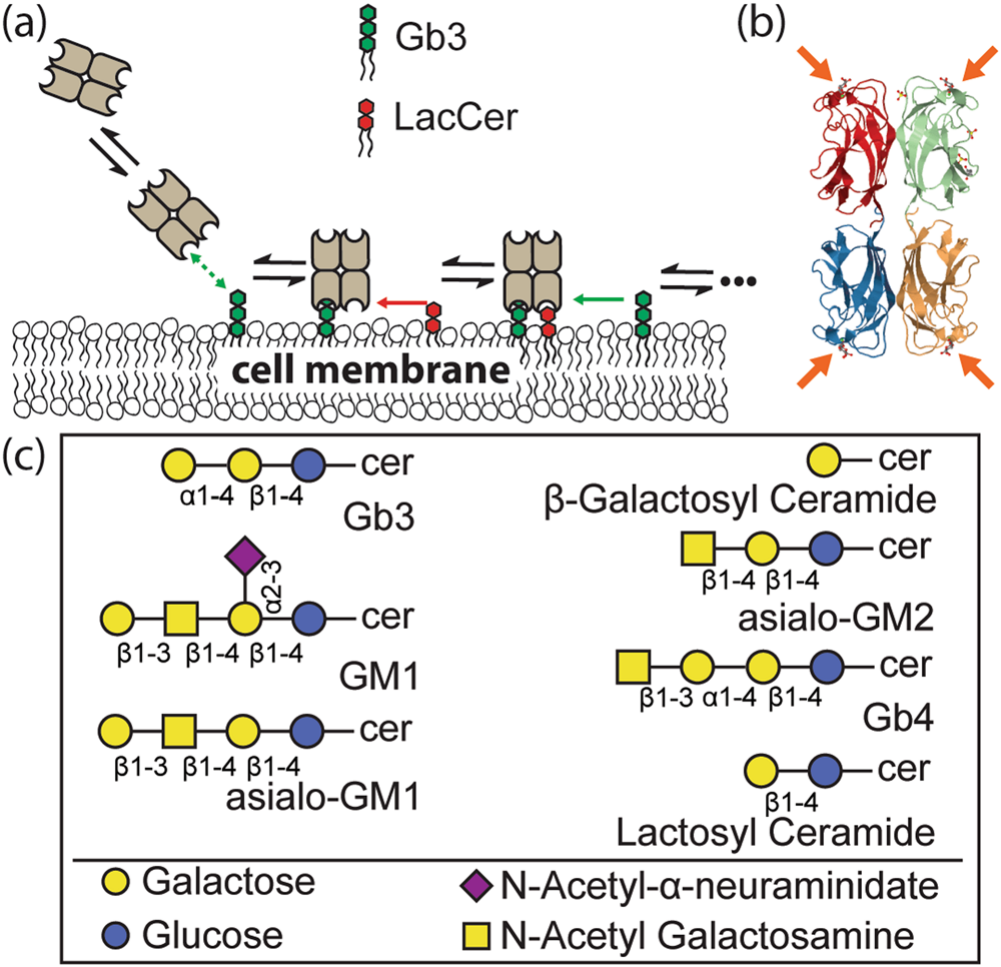
Schematic for the Reduction of Dimensionality (RD) model. (**a**) A schematic representation of RD influencing LecA interactions with the cellular membrane. LecA first diffuses from solution to a membrane surface and attaches to the high-affinity ligand, Gb3. Then, free membrane ligands move two dimensionally, enabling subsequent binding. The reduced dimensionality of diffusion enhances the effective concentrations of membrane ligands; thus, a weak ligand, such as LacCer, can contribute to LecA binding. (**b**) Graphical representation of LecA complexed with galactose as observed in the crystal structure (PDB code 1OKO)^19^. Four binding sites are indicated by arrows. Protein and carbohydrate are displayed in a cartoon representation with coloring done by subunit using JSmol. (**c**) Cartoon representations of glycolipids used on this study.

In this article, we first focus on LecA, a homotetrameric lectin, where each monomer has a single glycan binding site^17^. LecA contains two adjacent binding site pairs facing in opposite directions. (Figure 1) This configuration allows adhesion of *P. aeruginosa* to epithelial cells and may also contribute to linkages between bacteria, subsequently leading to biofilm formation^9,18^. It is known that LecA prefers binding to α-galactose terminated glycolipids; typically, globotriaosylceramide (i.e. Gb3, Galα1-4 Galβ1-4 Glc ceramide) is considered a major ligand for LecA^17,19–24^. However, it is known that LecA can bind to other types of glycolipids (e.g. β-galactose (Galβ) and N-acetylgalactosamine (GalNAc) terminated glycolipids), but the binding affinities are lower than with Gb3^22,25^_._

We recently reported a hetero-multivalent binding phenomenon for cholera toxin subunit B (CTB) in an environment that mimics the natural cell membrane^26,27^. Interestingly, we found that strong binding ligands could activate weak binding ligands via a fundamental mechanism, Reduction of Dimensionality (RD)^26^. We have illustrated the concept of RD in Fig. 1. The reaction rates of the subsequent binding events on the membrane surface are at least 10^4^ times higher than the first binding event^26^. Thus, even a weak binding ligand can participate in the second or higher order binding events resulting in higher protein attachment. This intrinsic mechanism suggests that the binding of multivalent proteins is not simply controlled by a single type of ligand; instead, the cooperative actions between strong and weak ligands can greatly influence the overall attachment of proteins and bacteria.

We hypothesized that the RD mechanism plays a key role in *P. aeruginosa* adhesion by influencing many different multivalent proteins, including LecA. Although Gb3 is the major LecA ligand, Gb3 is at low levels in human intestinal epithelial cells and murine lungs^28,29^. We suspected that Gb3 can activate abundant but weaker glycolipid ligands, influencing LecA attachment via the RD mechanism. We examined hetero-multivalency in LecA binding through analysis of hetero-multivalent binding cooperativities between major and minor LecA binding ligands. We were excited to find that high-affinity ligands were able to activate weak binding ligands, leading to positive hetero-multivalent cooperativity. Moreover, we designed a high-affinity liposome containing mixed ligands to target *P. aeruginosa* using the concept of the RD mechanism. Our study suggests that the inherent RD mechanism may play an essential role in various multivalent recognition systems.

## Results

**Figure 2:**
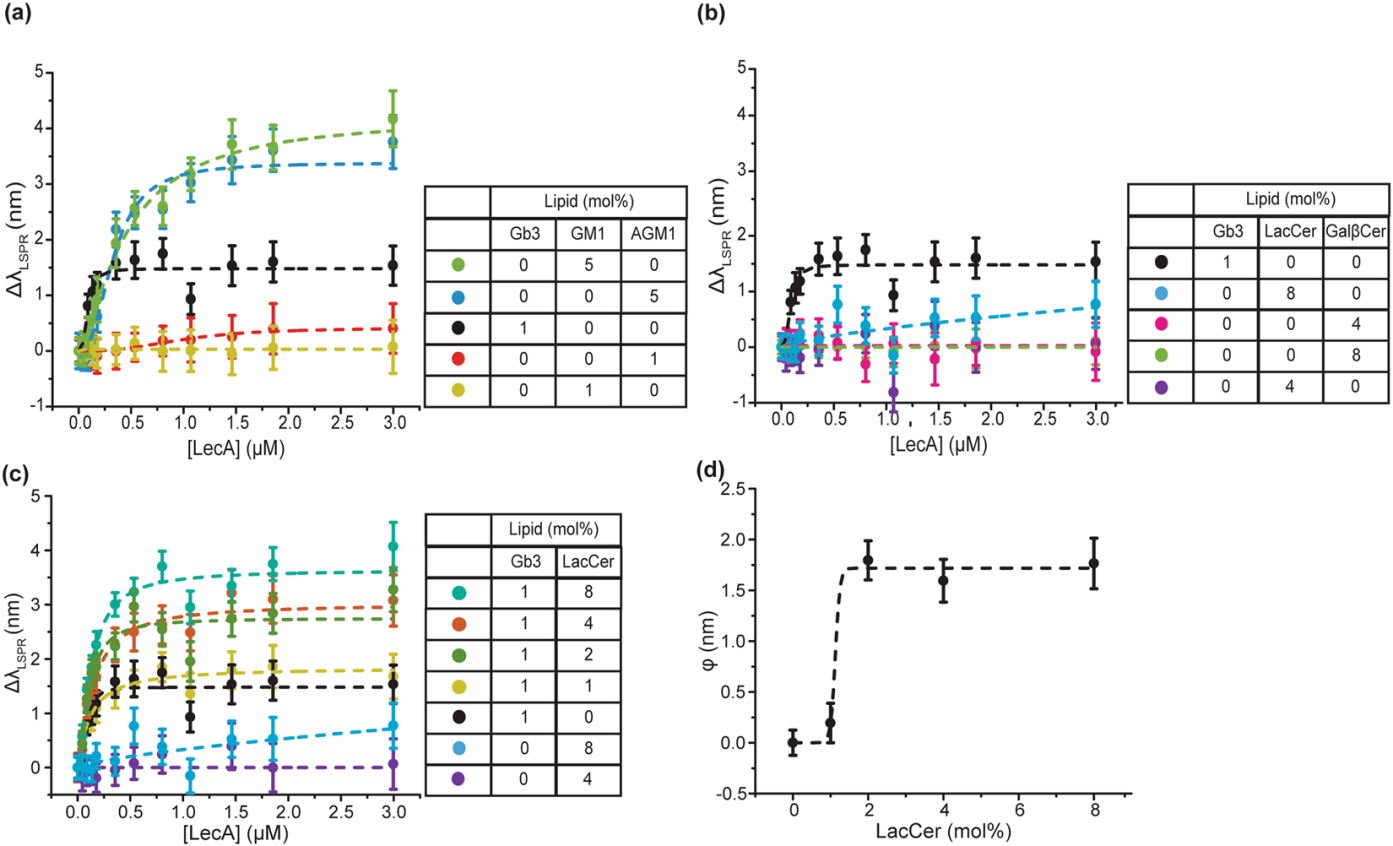
Saturation binding curves of LecA binding to common galactose terminated glycolipids and Gb3/LacCer mixtures that show positive cooperativity. The saturation binding curves’ dash lines represent the curve fits to Hill**’**s equation, fitted parameters are listed in SI Table 4. Data points are reported as mean ± S.D (n = 8). To better show the data points at low concentrations, the same binding curves on a semi-log scale are shown in the supplementary information. (**a** and **b**) Saturation binding curves of LecA binding to bilayers of common galactose terminated glycolipids. (**c**) Saturation binding curves of LecA binding to bilayers containing Gb3/LacCer mixtures. (**d**) ***ϕ*** values for 1 mol% of Gb3 mixed with different densities of LacCer. Dash line representing the fit of ***ϕ*** to the sigmoidal function is a guide to the eye.

Prior studies have shown that the presentation of glycan, such as oligosaccharides in solution, oligosaccharides on glycoarray surface, or glycolipids in cell membranes, can dramatically change the LecA binding^21,22,30^. In the glycoarray and glycolipid binding studies, LecA’s preferred ligand is known to be Gb3, but LecA can also bind to βGal terminated glycans^22,25,30–33^. To confirm the LecA binding affinities to different glycolipids, we first measured LecA binding to the common galactose-terminated glycolipids using the nanocube sensing platform (Fig. 2a,b and SI Fig. 4). LecA significantly bound to the bilayer containing 1 mol% Gb3. At the same density, LecA-AGM1 and LecA-GM1 binding was much weaker. When we increased the glycolipid density to 5 mol%, LecA binding to both AGM1 and GM1 became significant. LacCer and GalβCer were observed to be highly abundant in intestinal epithelium, and LacCer was noted as abundant in murine lungs^28,29^. However, their binding avidities are much lower than GM1 and AGM1. We could not observe LecA binding to LacCer surfaces unless the LacCer density was increased to 8 mol%. For GalβCer, LecA binding is still not measureable at an 8 mol% surface density. Based on these results, we rank the glycolipids in order of affinity, Gb3 $\gg$ GM1 ≈ AGM1 $\gg$ LacCer > GalβCer, and categorize them into three groups: (1) Strong ligands: Gb3; (2) Moderate ligands: GM1 and AGM1; (3) Weak ligands: LacCer and GalβCer.

### Positive binding cooperativity between strong and weak ligands (Gb3 and LacCer)

Based on the RD model, we expected that strong ligands would activate weak ligands, leading to higher binding capacity for LecA. To demonstrate this concept, we first measured LecA binding to the mixtures of Gb3 and LacCer. Keeping the density of Gb3 in the bilayer fixed at 1 mol%, we performed telescoping concentrations of LacCer in the bilayer (Fig. 2c and SI Fig. 5). LecA binding to pure 4 mol% surface density of LacCer was not measureable, and the binding at the highest LecA concentration (3 μM LecA) to pure 8 mol% LacCer is minimal. (Figure 2b and SI Fig. 4) After mixing LacCer with 1 mol% of Gb3, LecA binding to mixtures of Gb3 and LacCer was significantly higher than LecA binding to 1 mol% of Gb3. We can use hetero-multivalent cooperativity (ϕ in SI equation (2)) to quantify the enhanced binding capacity. In Fig. 2c, no obvious positive cooperativity was observed when 1 mol% Gb3 was mixed with 1 mol% LacCer, but cooperativity drastically increased at 2 mol% of LacCer. This result seems indicating that the surface density of the weak ligand has to reach a threshold value in order to contribute in LecA binding.

In addition to the threshold density of the weak ligand, we identified a second threshold of LecA concentration. SI Fig. 1 shows the changes in cooperativity at different LecA concentrations. The average cooperativity is minimal below 0.1 μM LecA but then increases until beginning to level off around 2 μM LecA. In the RD model, LecA has to first anchor to Gb3 in order to change from 3-D to 2-D diffusion, leading to an increased effective concentration of the weak ligand for the subsequent binding events. Thus, this hetero-multivalent binding process is limited by the first binding step, which corresponds to the dissociation constant of Gb3 (0.1 μM). This is probably the reason why the observed cooperativity significantly increased above the dissociation constant.

### Explore the RD Mechanism Using Kinetic Monte Carlo (kMC) Simulation

**Figure 3:**
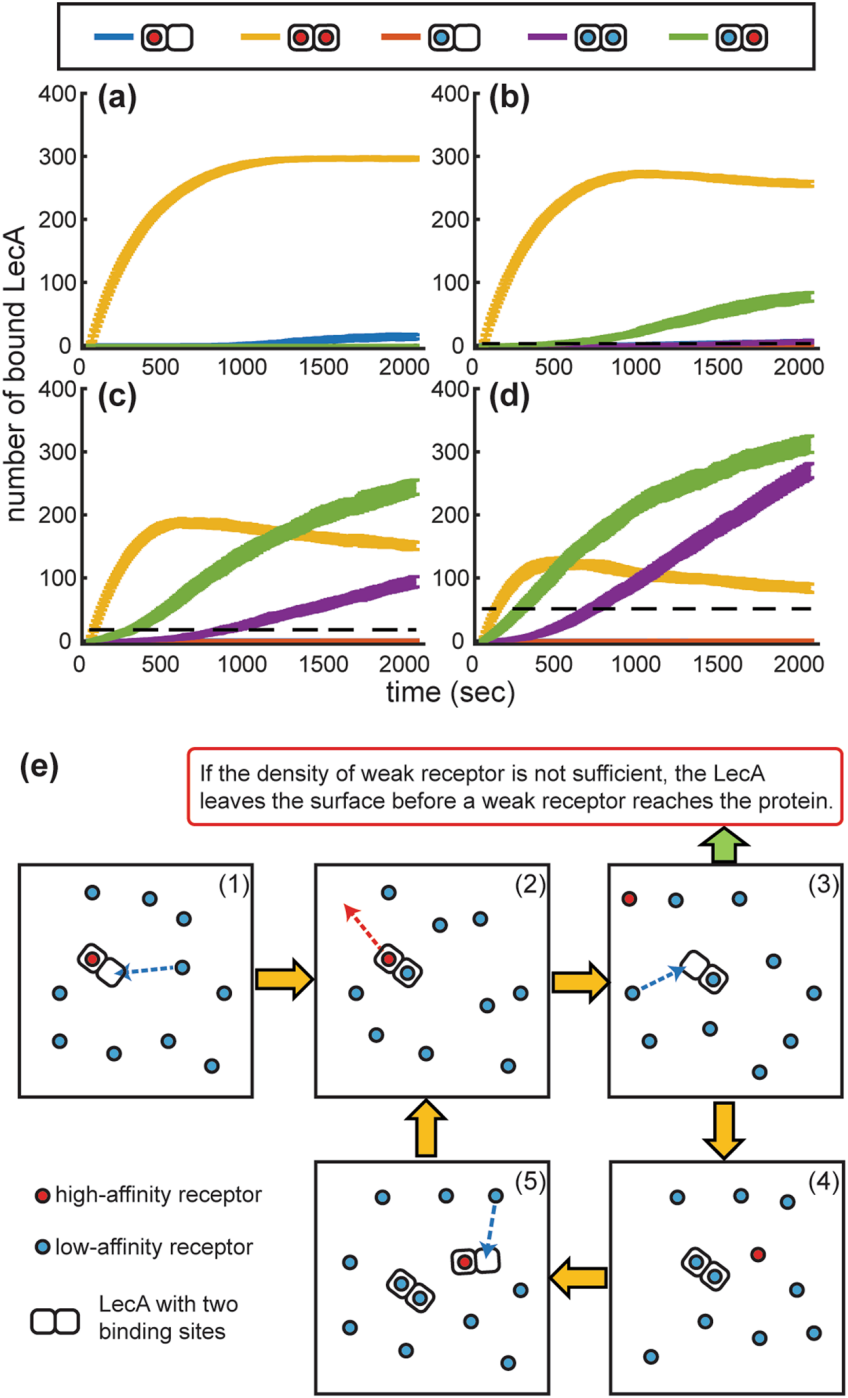
Modeling LecA binding kinetics using kMC simulation. LecA binding to a membrane surface containing 1 mol% of high-affinity ligands and various low-affinity ligand densities, (**a**) 0 mol%, (**b**) 0.5 mol%, (**c**) 3 mol%, and (**d**) 9 mol%. The affinity of the low-affinity ligand is 300-fold lower than the high-affinity ligand. (${{\boldsymbol{K}}}_{{\boldsymbol{d}},{\boldsymbol{low}}}=300{{\boldsymbol{K}}}_{{\boldsymbol{d}},{\boldsymbol{high}}}$ where ${{\boldsymbol{K}}}_{{\boldsymbol{d}}}={{\boldsymbol{k}}}_{-1}/{{\boldsymbol{k}}}_{1}$) Each curve represents the number of bound LecA in different binding configurations. The dashed line shows the maximum number of bound LecA at 2000 s without the high-affinity ligand at the same membrane density of low-affinity ligand. All data represented as average ± S.D from 50 kMC simulations. (**e**) A binding mechanism observed in the kMC simulation when the low-affinity ligand density is higher than the high-affinity ligand. (1) A LecA molecule moves from the solution phase to the membrane surface, and attaches to a high-affinity ligand. Then, a low-affinity ligand encounters the bound LecA completing the hetero-multivalent binding. (2) The high-affinity ligand dissociates from the bound LecA. (3) LecA binding to one low-affinity ligand is relatively unstable. At sufficient density, a low-affinity ligand can reach the free binding site before the LecA dissociates from the surface. (4) LecA binding to two low-affinity ligands is relatively stable. (5) The high-affinity ligand can facilitate the binding between LecA and low-affinity ligands by continuing the same process. (The figure shows only two binding sites that are participating in reactions happening on the surface. The other two binding sites facing in the opposite direction are not shown).

We hypothesized that the RD mechanism is the cause of the observed hetero-multivalency^26^. To further understand the influence of the RD mechanism, we performed a kMC simulation to model the stepwise binding of LecA. (Figure 3) The kMC simulation allows us to monitor the bound state of each individual LecA molecule; therefore, we can validate our hypothesis. The kMC simulation conducted on a two dimensional square with 250-by-250 square lattice sites (i.e. 212 × 212 nm^2^) represents the lipid bilayer. Glycolipid ligands are modeled as entities that can diffuse on a 2-dimensional membrane. Similar to the binding process shown in Fig. 1a, only two of the binding sites are exposed to one membrane surface at a time. Thus, the kMC simulation allows for two LecA binding sites attaching to and detaching from glycolipid ligands. The microscopic forward/reverse binding rate constants (*k*_1_ and *k*_−1_) between a high-affinity ligand (i.e. Gb3) and a LecA were estimated using literature values (parameter selection is described in the Supplementary information). The density of the high-affinity ligand was fixed at 1 mol%, and the density of the low-affinity ligand was varied from 0.5 to 9 mol%. The rate constants of low-affinity ligands were defined by reducing the forward rate constants of the high-affinity ligand 100-, 300-, and 1000-fold (SI Fig. 2).

In most cases, we observed ~90% of bound LecA attaching to two ligands. Due to reduced dimensionality of diffusion, the frequency of a ligand encountering a bound LecA dramatically increases; thus, LecA could rapidly find a second ligand on the membrane surface and complete the second binding. When a membrane contained strong ligands without weak ligands (Fig. 3a), the number of total bound LecA reached an equilibrium at ~1000 s. When 0.5 mol% of the weak ligands were mixed with 1 mol% of the strong ligands (Fig. 3b), hetero-multivalent binding occurred. Initially, the majority of LecA bound to two strong ligands. After the density of the unbound high-affinity ligand was reduced to one-third of the density of the unbound low-affinity ligand (~500 s), we could observe a significant portion of the low-affinity ligands contributing to LecA binding, leading to the increased binding capacity. Obviously, when the densities of the low-affinity ligands were raised (Fig. 3c,d), the low-affinity ligands could participate in LecA binding at an early time point.

Most surprisingly, we also observed a significant number of LecA molecules simultaneously binding to two low-affinity ligands. Without the high-affinity ligand, we could not observe the same number of LecA binding to the bilayer at the same densities of low-affinity ligands. Figure 3e shows the mechanism behind this phenomenon. A high-affinity ligand initiates attachment of LecA to the membrane surface; then, LecA can bind to an additional ligand or exchange bound ligands on the 2D membrane surface. However, a LecA molecule bound to only one low-affinity ligand will only maintain its bound state if it receives an unbound low-affinity ligand before the LecA molecule dissociates from the membrane.

It is obvious that the affinity of weak ligands can influence the hetero-multivalent binding process. (SI Fig. 2) When the affinity of weak ligands was decreased, the contribution of weak ligand to LecA binding reduced. For example, at 3 mol% density of weak ligand, weak ligands contributed 55%, 44%, and 31% of the LecA bound ligands for 100-, 300-, and 1000-fold reduced affinity, respectively. To enhance the contribution of the weak ligand, the density of weak ligand should be increased. This also corroborates our experimental observation that a threshold concentration of the weak ligand is required to enable its contribution in protein binding. Another noticeable phenomenon is that LecA binding to the mixed bilayer requires longer time to reach an equilibrium state. This is because the rearrangement of the bound ligands requires multiple stepwise reactions.

It is worth noting that the kMC simulation considers a simple two-step binding process without complex biological assumptions, such as ligand clustering, membrane curvature, or allosteric regulation. We still observed the same degree of hetero-multivalent binding cooperativity in the kMC simulation and the nanocube measurement, demonstrating the essence of the RD mechanism in hetero-multivalent binding systems.

### Influence of hetero-multivalency on LecA binding avidity

**Figure 4:**
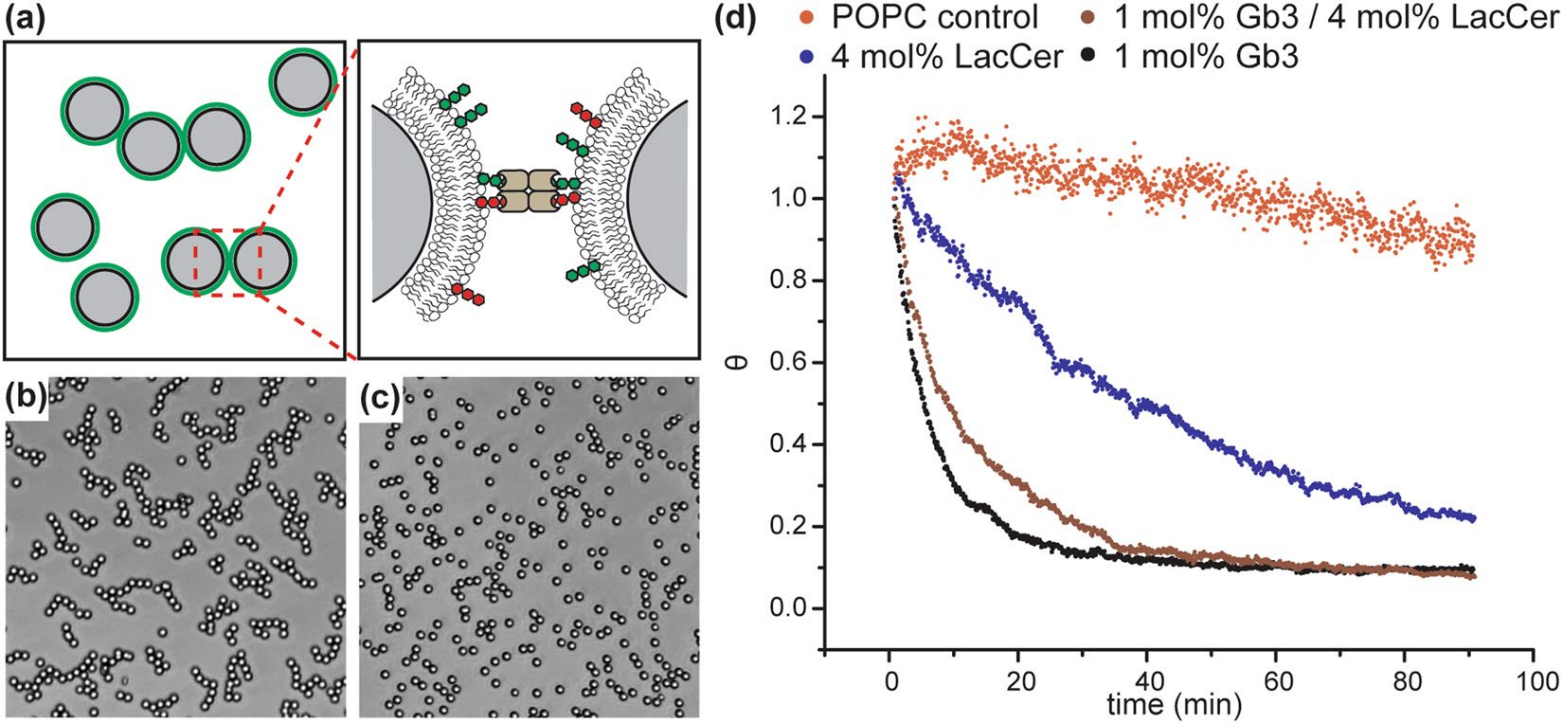
Colloid aggregation kinetics. (**a**) A schematic drawing of silica particle aggregation induced by LecA-glycolipid binding. (**b**) A snapshot of particle aggregation mediated by LecA tethering. (**c**) A snapshot of particle dispersion without LecA. (**d**) Particle aggregation at different conditions. The decay rate of the singlet ratio (θ) is associated with the binding avidity between LecA and membrane ligands.

According to the kMC simulation, we observed a portion of LecA binding to one or two LacCer after mixing Gb3 with LacCer. This phenomenon would decrease the average binding energy (avidity) between LecA and the bilayer. To confirm this argument, we used a nascent video microscopy technique developed by Duncan and Bevan to directly measure the binding avidity^34^. (Fig. 4 and Supplementary text S1) The calculated aggregation rate (*k*_11_) significantly decreased when LacCer was mixed with Gb3 in the lipid bilayer, indicating the binding avidity reduced in the Gb3/LacCer mixture. (SI Table 1) This phenomenon is consistent with the kMC simulation. When LecA interacts with pure 1 mol% Gb3 bilayers, a LecA could bind to maximum of four Gb3 ligands, forming a strong linkage between the silica beads. When LecA interacts with the Gb3/LacCer mixture, LacCer could compete with Gb3 ligands in LecA binding. In this situation, a portion of LecA might bind to both Gb3 and LacCer simultaneously, leading to a weaker linkage between two silica beads.

### Hetero-multivalency between strong/moderate/weak ligands

**Figure 5:**
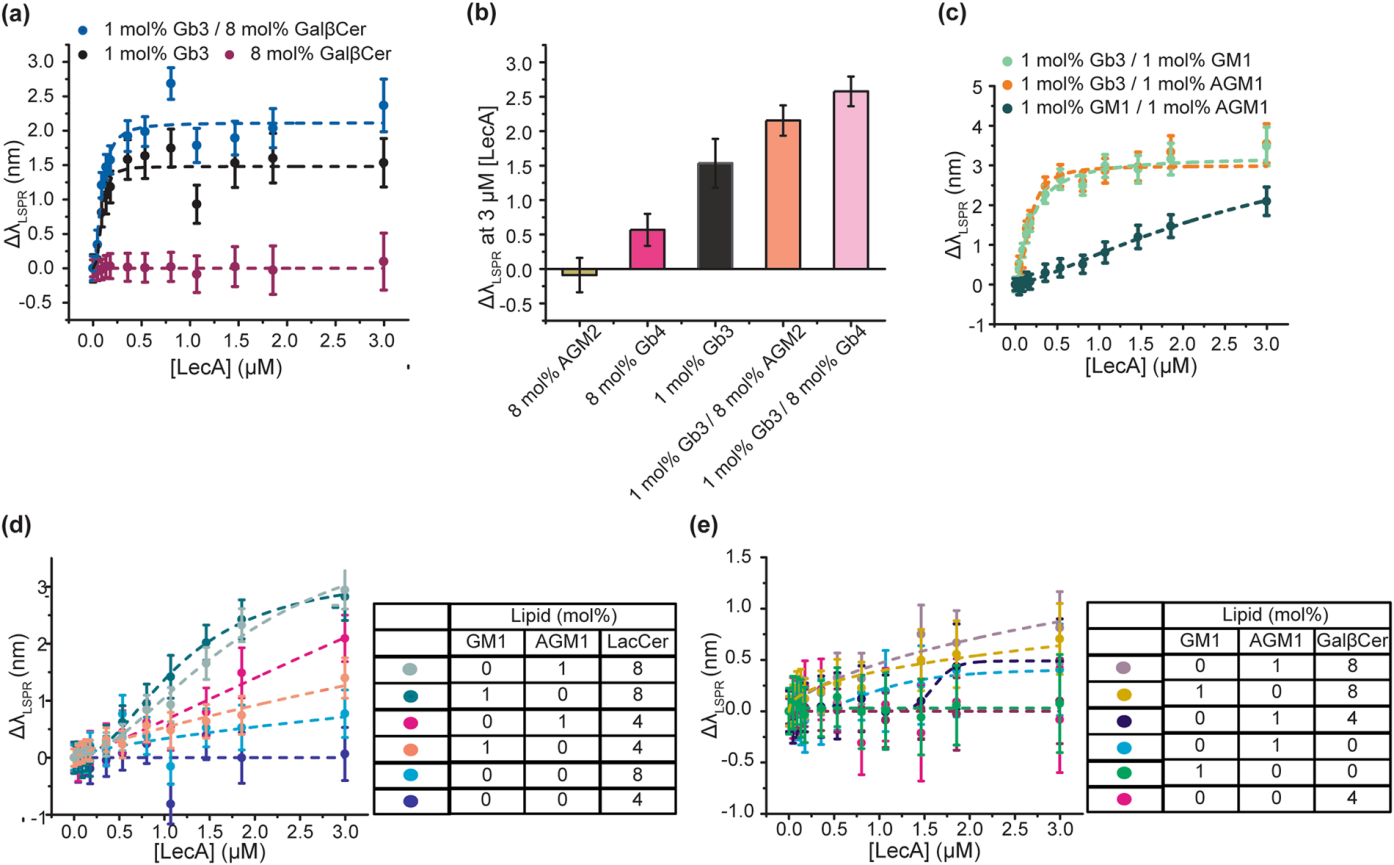
Cooperativity between strong, moderate, and weak ligands. (**a**) Binding curves of LecA to the mixture of Gb3/GalßCer. (**b**) 1 mol% Gb3 mixed with GalNAc terminated glycolipids at 3 μM LecA. (**c**) Binding curves of LecA to the mixture of the strong (Gb3)/moderate (GM1 or AGM1) ligands. (**d**) Binding curves of LecA to the mixture of the moderate (GM1 or AGM1) and LacCer. (**e**) Binding curves of LecA to the mixture of the moderate (GM1 or AGM1) and GalβCer. All data points are reported as mean ± S.D (n = 8). The dashed lines represent Hill equation fits to the data.

Based on the RD mechanism, we expected to observe the same hetero-multivalent binding cooperativity between Gb3 and other glycolipid ligands. (Fig. 5) We first mixed Gb3 with the simplest glycolipid, galactosylceramide (GalβCer), which consists of a single β–galactose residue. GalβCer is highly abundant in the brain and intestinal epithelial cells^29,35^; thus, it may play a role in the LecA binding process. As expected, we observed positive cooperativity when 8 mol% GalβCer was mixed with 1 mol% Gb3 (Fig. 5a, SI Fig. 6 and SI Table 2). However, the degree of enhancement is lower than the Gb3/LacCer combination. This is probably because the binding affinity of GalβCer is weaker than LacCer, as is observed in kMC simulations.

LecA also has weak binding affinity to N-acetylgalactosamine (GalNAc) terminated glycans^21,22,25^. Thus, we also evaluated the hetero-multivalency between Gb3 and N-acetylgalactosamine (GalNAc) terminated glycolipids, Gb4, and AGM2 (Fig. 5b, Supplementary text S2). Again, the result shows that Gb3 could form a partnership with GalNAc terminated glycolipids, leading to positive cooperativity. Obviously, the moderate ligands (AGM1 and GM1) could also be activated via the same RD mechanism (Fig. 5c). In addition to testing cooperativity between Gb3 and moderate ligands, we compared the cooperativity amongst the moderate ligands themselves. We observed much greater LecA attachment in the GM1/AGM1 mixture. The increase of available ligands in the lipid bilayer is probably the reason for the increased cooperativity among moderate ligands.

In addition, we wondered if the moderate ligands (AGM1 and GM1) were sufficient to activate weak ligands, leading to higher LecA attachment. First, we investigated the binding cooperativity between LacCer and the moderate ligands (Fig. 5d and SI Fig. 5). We observed positive cooperativity between 1 mol% of each moderate ligand, individually, and 4 or 8 mol% of LacCer. This observation indicated that the moderate ligands were able to activate LacCer. We also examined the change of cooperativity at different LecA concentrations (SI Fig. 1). Similar to Gb3/LacCer system, the cooperativity became significant when the LecA concentration reached a threshold value. However, the threshold concentration of the moderate ligands (~0.5 μM) was higher than the threshold of Gb3 (0.1 μM). As discussed above, the threshold of LecA concentration is probably dominated by the first binding step, which is associated with the dissociation constant of the ligand with higher affinity. Thus, we observed cooperativity significantly increased after the LecA concentration reached the dissociation constants of the moderate ligands (~0.5 μM).

The cooperativity between the moderate ligands and GalβCer is not as significant as LacCer (Fig. 5e, SI Fig. 6 and Supplementary text S3). Regardless, we have confirmed that LecA binding capacity enhancement is not necessarily limited to just the highest affinity ligand, Gb3, but can also be observed with GM1 and AGM1 mixtures. This is similar to the case with CTB in which positive cooperativity is observed with GM1 (a strong ligand), fucosyl-GM1(moderate), and GD1b (moderate)^26^.

### Hetero-multivalency between liposome and bacterium

A key concept of the RD mechanism is that a strong ligand can activate weaker ligands, resulting in enhanced ligand binding. We observed this binding enhancement with two different bacterial lectins, LecA and CTB^26,27^. The same mechanism may occur in other types of multivalent binding systems, such as bacteria and viruses. We wondered if we could utilize the RD mechanism to design a high affinity liposome for targeting bacteria. A bacterium can have multiple surface adhesins that can bind to various host cell ligands with different affinities. Therefore, some ligands may exhibit relatively low binding affinities to bacterial adhesins. If we are able to fabricate a liposomal drug carrier containing both high- and low-affinity ligands, a liposome can simultaneously attach to multiple different surface adhesins in a bacterium, leading to higher retention of the drug carrier.

**Figure 6:**
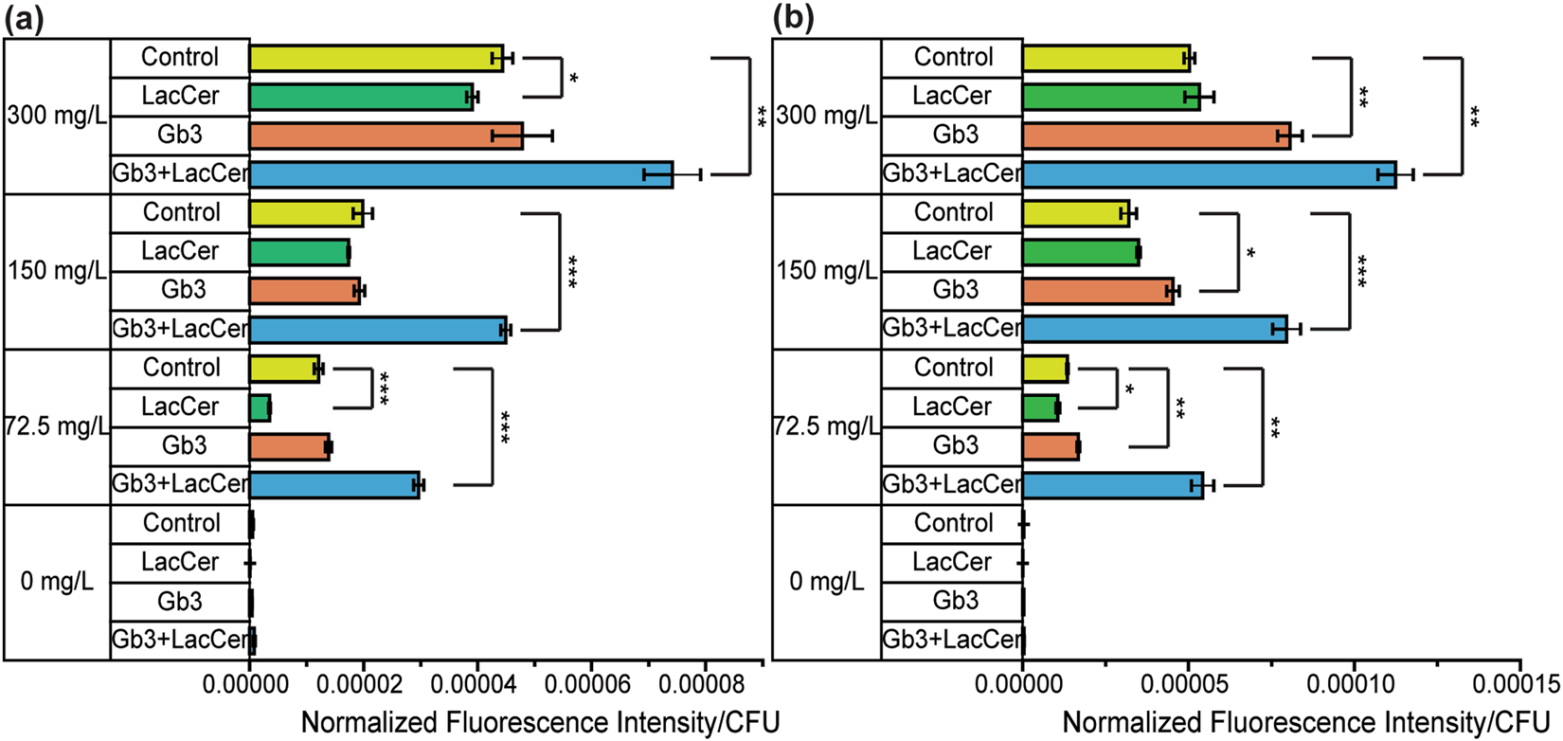
Liposome binding to *P. aeruginosa*. Retention of fluorescent liposomes on *P. aeruginosa* ((a) PAO1 and (**b**) Xen41) was quantified by normalized fluorescence intensity per colony forming unit (CFU). The liposome concentration given is mass concentration. Control (yellow) is 99.5 mol% POPC/0.5 mol% TR-DHPE. LacCer (green) is 10 mol% LacCer/89.5 mol% POPC/0.5 mol% TR-DHPE. Gb3 (orange) is 10 mol% Gb3/89.5 mol% POPC/0.5 mol% TR-DHPE. Gb3/LacCer (blue) is 5 mol% LacCer/5 mol% Gb3/89.5 mol% POPC/0.5 mol% TR-DHPE. The error bars are standard deviation (n = 3). The stars indicate t-test unequal variance p-values of p < 0.1 (*), p < 0.05 (**), and p < 0.01 (***). (SI Table 5).

We fabricated fluorescent liposomes containing 10 mol% Gb3, 10 mol% LacCer, and an equal parts combination of the two (5 mol% Gb3/5 mol% LacCer) to target *P. aeruginosa*. As discussed above, Gb3 is a strong ligand, and LacCer is a weak ligand for LecA. Prior literature also reported that T4P of *P. aeruginosa* could attach to β-Gal terminated glycans^10,36^. Thus, we expected LacCer could serve as a ligand for both LecA and T4P. The control liposome contained only POPC lipid. The composition of control liposomes is similar to the formulation of liposomal antibiotics currently in phase 3 clinical trials^37–39^. We evaluated liposome targeting efficiencies in binding to two *P. aeruginosa* strains, PAO1 and Xen41, by measuring the retention of liposomes by the bacteria. The normalized fluorescence results of binding liposomes to 48 hour cultured bacteria are shown in Fig. 6.

The retention of the liposomes containing 10 mol% of LacCer was not higher than the control liposome. The retention of 10 mol% of Gb3 was slightly higher than the control system, but the difference varied insignificance. Interestingly, for Gb3/LacCer liposomes (5 mol% + 5 mol%), the retention was significantly greater than the other liposomal formulations tested. Compared to the control system, the retention of Gb3/LacCer liposomes was enhanced up to 4-fold (for Xen41, 2.5-fold for PAO1) at the lowest liposome concentration (0.0725 g/L). Because the formula of the control liposome is similar to clinical liposomal antibiotics, this result indicated that we can improve the current drug formula by simply introducing two host cell molecules. These demonstrate the potential to use mixed host cellular ligands to improve liposomal targeting of *P. aeruginosa*.

## Discussion

Recent research on multivalent binding has suggested that total and relative densities of glycotopes in heterogeneous environment has an impact on carbohydrate-protein recognition events and cannot be explained by the simple on-off switch model^40^. In this paper, we have investigated LecA binding in heterogeneous glycolipid environment. Mixing high-affinity ligands with weakly binding ligands could alter the LecA binding behavior. The kMC simulations and experimental results indicated that the changes of binding capacity and avidity are probably induced by the RD mechanism. In order to initiate cooperative binding, we found two conditions must be satisfied. First, there is a minimum LecA concentration required before observing significant cooperativity. The minimum concentration corresponds to the dissociation constants of the highest affinity ligands present in the model membrane. This criterion is predicted by the RD mechanism. In the RD mechanism, the first binding event brings a ligand from the solution phase to the model membrane; then, the effective ligand concentrations increase for the subsequent binding events due to the reduced dimensionality of diffusion. Therefore, the occurrence of hetero-multivalent binding is limited by the first binding event, which corresponds to the dissociation constant between LecA and the highest affinity ligand.

The second criterion is that a sufficient amount of the weaker ligand is required to trigger hetero-multivalency. Through the analysis of the kMC simulation, the retention rate of LecA by the weak ligand and the frequency that weak ligands encounter membrane-bound LecA are the two key parameters that determine the degree of hetero-multivalent binding. Thus, this threshold density is associated with the affinity of the weaker ligand. For Gb3/LacCer mixture, no obvious cooperativity was observed at 1 mol% of LacCer, but the cooperativity drastically increased at 2 mol% of LacCer. When Gb3 was mixed with the moderate ligands (GM1 & AGM1), we observed significant cooperativity at 1 mol% of the moderate ligand. The same trend was observed in the kMC simulation. When the affinity of the weak ligand is reduced, a higher density of the weak ligand is required to observe the participation of weak ligand in LecA binding.

The threshold density of LacCer, approximately 2 mol%, is a noticeable portion of the total model membrane. This raises the question of whether LacCer in epithelial cells is present in sufficient quantities to play a role in LecA binding. To address this concern, we note that glycolipids are highly enriched in the apical plasma membrane of polarized epithelial cells^41–43^. Additionally, it has been shown that the glycolipid content can reach up to 30% of the total membrane lipids in microvilli^44^. This is significant as the typical total glycolipid fraction of the entire membrane for mammalian cells is ~5%^45^. Furthermore, Parkin *et al*. observed the microvillar membranes in porcine kidney cortex contain 3.53 mass% of LacCer, and LacCer was further enriched up to 7.26 mass% in detergent-resistant domains of microvilli^46^. Besides cell polarization, Gb3 can also cluster with galactosyl ceramide, glucosyl ceramide, and LacCer in cholesterol enriched domains^47^. These clustering processes could further concentrate local glycolipid abundance. Therefore, it is reasonable to expect that the threshold density of LacCer is biologically relevant on a local scale. In addition, we expect that the localized enrichment of membrane ligands induced by phase separation, dynamics of the cell cytoskeleton, cell polarization, and lipid asymmetry can influence the effect of the RD mechanism. Further studies are required to dissect the role of the RD mechanism in biological systems.

It should be noted that binding capacity (total amount of bound proteins) is not directly correlated with binding avidity (total binding energy between a protein and ligands) in multivalent binding systems. According to the kMC results, strong ligand can facilitate LecA binding to weak ligands, resulting in increased binding capacities. In the same situation, a significant portion of LecA can bind to both Gb3 and LacCer ligands or to two LacCer ligands; therefore, we expect that the binding avidity would be lower than that of LecA binding to two Gb3 ligands. The changes of binding capacity and binding avidity may affect downstream processes of LecA. For instance, Eierhoff *et al*. showed that LecA-Gb3 interaction is critical to induce *P. aeruginosa* invagination of giant unilamellar vesicles (GUVs) and H1299 cells^16^. Their experimental data demonstrated the threshold density of Gb3 to be 0.1 mol% for bacterial engulfment which is much higher than the Gb3 content in lung epithelium. Based on their theoretical model, a higher number of LecA-Gb3 binding events and higher adhesion energy can enhance membrane engulfment of *P. aeruginosa*. Therefore, it is reasonable to hypothesize that the potential hetero-multivalent binding of LecA influences the invagination process. Another example is that Gb3 serves as a signaling ligand for LecA to induce CrkII phosphorylation^11^. The participation of weak ligands, such as LacCer, may change the LecA-Gb3 interactions, altering the signaling response. Additionally, it has also been reported that ligands binding to LacCer can activate Src family kinase Lyn^48^. Thus, the hetero-multivalent binding of lectins may introduce a possible secondary role of lectins in the Lyn signaling pathway. Further investigation is required to understand the potential role of hetero-multivalency in various biological systems.

Besides demonstrating a LecA binding mechanism, we showed the potential of using hetero-multivalent binding to improve targeted drug delivery. Traditionally, targeted drug delivery schemes have tended to decorate the drug carrier with the highest affinity ligands^49,50^; however, this strategy often leads to higher off-target binding. A recent computational study suggests that using a combination of multiple weaker affinity ligands can improve selectivity, and that selectivity can be further optimized by varying the ligand surface densities^51^. This theoretical study brings light to a new aspect of targeted drug delivery. However, using a set of low affinity ligands may reduce the targeting efficiency of drug carriers. A potential solution is to decorate weak-affinity ligands on fluidic liposome surfaces along with a moderate ligand that can facilitate weak ligand-ligand binding via the RD mechanism. Thus, we believe liposomal carriers are an attractive approach for the design of multivalent-targeted drug delivery systems.

Our liposome-bacterium studies demonstrated the applicability of glycolipid mixtures to achieve improved liposome targeting to *P. aeruginosa*. Specifically, our results yielded two main conclusions. First, adding multiple types of glycolipids can significantly improve liposome binding beyond single glycolipid liposomes. Given the observed binding pattern, LecA is probably not the only actor at work in liposome binding to *P. aeruginosa*. We believe other galactose binding adhesins, such T4P, contribute to the observed liposome targeting. Second, the binding between *P. aeruginosa* and liposomes containing only LacCer ligand was negligible. Therefore, LacCer has to form a partnership with Gb3 ligand in order to exhibit improved liposome retention. This phenomenon is consistent with the LecA and CTB binding systems. Weak ligands need the assistance of high-affinity ligands to initiate hetero-multivalent binding. This phenomenon presents an issue to conventional ligand-ligand screening assays (e.g. microarray technology) because they screen ligands one by one. As a result, conventional methods may miss the essential weak binding ligands, which could exhibit high binding selectivity to the target pathogens. Thus, our previously published membrane perturbation protocol could provide a more efficient strategy to screen potential weak ligands involving *P. aeruginosa* binding^26^. In summary, the proof-of-concept liposome-targeting test has demonstrated the application of a hetero-multivalent targeting strategy. However, there is much work to be done to create a rational basis for *a priori* targeting design in terms of both affinity and selectivity.

## Conclusion

RD is an intrinsic mechanism that seemingly occurs in all multivalent binding processes. The low-affinity ligands can also contribute to the binding process via this simple mechanism. As such, the high-affinity molecule is not the only ligand to consider in multivalent binding processes; the multivalent recognition is determined by the cooperativity among high-affinity and low-affinity ligands. The simple RD mechanism adds another level of complexity to biological systems. Further studies are required to dissect the role of the RD mechanism in various biological systems. Besides LecA binding, we also demonstrated the application of hetero-multivalency to target the whole bacteria. Our preliminary studies demonstrate the potential of improved efficiency in targeted drug delivery.

## Methods

The materials and methods are detailed in Supplementary Information.

### Nanocube Synthesis and Supported Lipid Bilayer Formation on Ag@SiO2 Nanocubes

The nanocube synthesis procedure is originally from Tao *et al*.^52^. The silica coating procedure was originally described in Wu *et al*.^53^ and modified in Worstell *et al*.^27^. Supported lipid bilayers were formed on the nanocubes using a modified vesicle fusion method^27^.

### Supported Lipid Bilayer Formation on Silica Beads

Small unilamellar vesicles (SUVs) were prepared via extrusion (Supplementary Information)^27^. Supported lipid bilayers were formed on the silica beads using a vesicle fusion method^54^. Then, the beads were blocked with BSA for 1 hour.

### Nanocube Protein Binding Measurement

Bilayer coated nanocubes were incubated for 1 hour with BSA to reduce nonspecific binding. Then, the desired amount of LecA was added and the test, control, and blank solutions were pipetted into a 384 well plate. The plate was read using a UV/Vis microplate reader spectrophotometer equipped with a CCD. The change in the location of the quadrupole LSPR (Localized Surface Plasmon Resonance) peak (LSPR peak) can be correlated to the amount of LecA attached to the bilayer.

### Video Microscopy for Silica Particle Aggregation

Wells of a 96 well-plate were coated with polyethylene glycol (PEG) using Pluronic F-127. The procedure for video microscopy was adapted from Duncan *et al*.^34^. LecA solution was added to a PEG-coated well followed by bilayer coated silica beads and images were collected using an inverted optical microscope with a 20x objective. An image analysis algorithm was used to locate and track centers of each particle^55,56^.

### *P. aeruginosa* Liposomal Targeting

*P*. *aeruginosa* strains PAO1/pJDC233 and Xen41 were cultured overnight in LB medium at 37 °C and incubated in 96 well plates at 37 °C for 48 hours. Planktonic cells were carefully pipetted out and attached cells were washed with TBS. Then, Gb3, POPC, LacCer or Gb3/LacCer liposomes were added and incubated at 37 °C for 2 hours. The fluorescent signals of the liposome bound bacteria were detected using fluorescent spectrophotometer at an Excitation/Emission wavelength of 580 nm/620 nm. Bacterial enumeration was performed by plating on solid media to establish bacterial cell count (CFU/mL).

### Statistical Analysis

All data are represented as mean ± standard deviation (S.D.). The Hill-Waud model was fit to binding curves via the Levenberg Marquardt algorithm. Welch’s unequal variances t-test was applied to the P. aeruginosa liposomal binding data.

### Kinetic Monte Carlo (kMC) Simulation

The kMC algorithm was implemented to model the kinetics of LecA binding to a membrane containing both high-affinity and low-affinity ligands^57,58^. The surface of lipid bilayer is modelled as a 250-by-250 square lattice sites (i.e. 212 × 212 nm^2^) with a periodic boundary condition, and ligands are randomly distributed on the surface.

### Data Availability

The kMC datasets have been provided in the supplementary data files. Other datasets generated during and/or analyzed during the current study are available from the corresponding author on reasonable request.

## Electronic supplementary material

Supplementary Information

SI Dataset 1

SI Dataset 2

SI Dataset 3

Videoset 1

Videoset 2

Videoset 3

## References

[1] Stryjewski, M. E. & Sexton, D. J. In *Severe Infections Caused by* Pseudomonas Aeruginosa (eds Alan R. Hauser & Jordi Rello) 1–15 (Springer US, 2003).

[2] Gellatly SL, Hancock RE (2013). Pseudomonas aeruginosa: new insights into pathogenesis and host defenses. Pathog Dis.

[3] Chi E, Mehl T, Nunn D, Lory S (1991). Interaction of Pseudomonas aeruginosa with A549 pneumocyte cells. Infect Immun.

[4] Fleiszig SM, Zaidi TS, Fletcher EL, Preston MJ, Pier GB (1994). Pseudomonas aeruginosa invades corneal epithelial cells during experimental infection. Infect Immun.

[5] Fleiszig SMJ (1996). Relationship between cytotoxicity and corneal epithelial cell invasion by clinical isolates of Pseudomonas aeruginosa. Infection and Immunity.

[6] Mewe M (2005). Pseudomonas aeruginosa lectins I and II and their interaction with human airway cilia. The Journal of laryngology and otology.

[7] Chemani C (2009). Role of LecA and LecB lectins in Pseudomonas aeruginosa-induced lung injury and effect of carbohydrate ligands. Infect Immun.

[8] Fong, J. N. & Yildiz, F. H. Biofilm MatrixProteins. *Microbiol Spectr* **3**, 10.1128/microbiolspec.MB-0004-2014 (2015).

[9] Diggle SP (2006). The galactophilic lectin, LecA, contributes to biofilm development in Pseudomonas aeruginosa. Environ Microbiol.

[10] Saiman L, Prince A (1993). Pseudomonas aeruginosa pili bind to asialoGM1 which is increased on the surface of cystic fibrosis epithelial cells. J Clin Invest.

[11] Zheng S (2017). The Pseudomonas aeruginosa lectin LecA triggers host cell signalling by glycosphingolipid-dependent phosphorylation of the adaptor protein CrkII. Biochim Biophys Acta.

[12] Funken H (2012). Specific association of lectin LecB with the surface of Pseudomonas aeruginosa: role of outer membrane protein OprF. PLoS One.

[13] Cott C (2016). Pseudomonas aeruginosa lectin LecB inhibits tissue repair processes by triggering beta-catenin degradation. Biochim Biophys Acta.

[14] Kühn K (2015). The interplay of autophagy and β-Catenin signaling regulates differentiation in acute myeloid leukemia. Cell Death Discov.

[15] Schneider D (2015). Lectins from opportunistic bacteria interact with acquired variable-region glycans of surface immunoglobulin in follicular lymphoma. Blood.

[16] Eierhoff T (2014). A lipid zipper triggers bacterial invasion. Proceedings of the National Academy of Sciences of the United States of America.

[17] Imberty A, Wimmerova M, Mitchell EP, Gilboa-Garber N (2004). Structures of the lectins from Pseudomonas aeruginosa: insights into the molecular basis for host glycan recognition. Microbes and Infection.

[18] Grishin AV, Krivozubov MS, Karyagina AS, Gintsburg AL (2015). Pseudomonas Aeruginosa Lectins As Targets for Novel Antibacterials. Acta naturae.

[19] Cioci G (2003). Structural basis of calcium and galactose recognition by the lectin PA-IL of Pseudomonas aeruginosa. FEBS Lett.

[20] Gilboa-Garber N, Mizrahi L, Garber N (1972). Purification of the galactose-binding hemagglutinin of Pseudomonas aeruginosa by affinity column chromatography using sepharose. FEBS Lett.

[21] Lanne B, Cîopraga J, Bergström J, Motas C, Karlsson K-A (1994). Binding of the galactose-specificPseudomonas aeruginosa lectin, PA-I, to glycosphingolipids and other glycoconjugates. Glycoconjugate Journal.

[22] Blanchard B (2008). Structural basis of the preferential binding for globo-series glycosphingolipids displayed by Pseudomonas aeruginosa lectin I. J Mol Biol.

[23] Chen CP, Song SC, Gilboa-Garber N, Chang KS, Wu AM (1998). Studies on the binding site of the galactose-specific agglutinin PA-IL from Pseudomonas aeruginosa. Glycobiology.

[24] Villringer S (2018). Lectin-mediated protocell crosslinking to mimic cell-cell junctions and adhesion. Scientific reports.

[25] Mahal, L. K., To generate specificity profiles for commercially available reagents for the community and to facilitate the comparison of glycan arrays on multiple platforms. *Glycan array data in Consortium for Functional Glycomics, dataset number: primscreen_4787, primscreen_4788, primscreen_4789, primscreen_4790*, **2011**. Available from: www.functionalglycomics.com.

[26] Krishnan P (2017). Hetero-multivalent binding of cholera toxin subunit B with glycolipid mixtures. Colloids and surfaces. B, Biointerfaces.

[27] Worstell NC, Krishnan P, Weatherston JD, Wu HJ (2016). Binding Cooperativity Matters: A GM1-Like Ganglioside-Cholera Toxin B Subunit Binding Study Using a Nanocube-Based Lipid Bilayer Array. PLoS One.

[28] Momoeda K (1996). Developmental changes of neutral glycosphingolipids as receptors for pulmonary surfactant protein SP-A in the alveolar epithelium of murine lung. J Biochem-Tokyo.

[29] Breimer ME, Hansson GC, Karlsson KA, Larson G, Leffler H (2012). Glycosphingolipid composition of epithelial cells isolated along the villus axis of small intestine of a single human individual. Glycobiology.

[30] Reynolds M, Marradi M, Imberty A, Penades S, Perez S (2012). Multivalent gold glycoclusters: high affinity molecular recognition by bacterial lectin PA-IL. Chemistry.

[31] Chabre YM (2011). Combining glycomimetic and multivalent strategies toward designing potent bacterial lectin inhibitors. Chemistry.

[32] Bernardi A (2013). Multivalent glycoconjugates as anti-pathogenic agents. Chemical Society reviews.

[33] Cecioni S (2012). Rational design and synthesis of optimized glycoclusters for multivalent lectin-carbohydrate interactions: influence of the linker arm. Chemistry.

[34] Duncan GA, Bevan MA (2014). Tunable aggregation by competing biomolecular interactions. Langmuir.

[35] Schnaar, R. L. & Kinoshita, T. In *Essentials of Glycobiology* (eds A. Varki *et al*.) (2015–2017). 27010055

[36] Comolli JC, Waite LL, Mostov KE, Engel JN (1999). Pili binding to asialo-GM1 on epithelial cells can mediate cytotoxicity or bacterial internalization by Pseudomonas aeruginosa. Infection and Immunity.

[37] Loira-Pastoriza C, Todoroff J, Vanbever R (2014). Delivery strategies for sustained drug release in the lungs. Adv Drug Deliv Rev.

[38] Cipolla D, Gonda I, Chan HK (2013). Liposomal formulations for inhalation. Ther Deliv.

[39] Meers P (2008). Biofilm penetration, triggered release and *in vivo* activity of inhaled liposomal amikacin in chronic Pseudomonas aeruginosa lung infections. The Journal of antimicrobial chemotherapy.

[40] Jimenez Blanco JL, Ortiz Mellet C, Garcia Fernandez JM (2013). Multivalency in heterogeneous glycoenvironments: hetero-glycoclusters, -glycopolymers and -glycoassemblies. Chemical Society reviews.

[41] Simons K, Toomre D (2000). Lipid rafts and signal transduction. Nat Rev Mol Cell Biol.

[42] van Meer G, Stelzer EH, Wijnaendts-van-Resandt RW, Simons K (1987). Sorting of sphingolipids in epithelial (Madin-Darby canine kidney) cells. J Cell Biol.

[43] van Meer G, Simons K (1986). The function of tight junctions in maintaining differences in lipid composition between the apical and the basolateral cell surface domains of MDCK cells. EMBO J.

[44] Danielsen EM, Hansen GH (2008). Lipid raft organization and function in the small intestinal brush border. J Physiol Biochem.

[45] Danielsen EM, Hansen GH (2006). Lipid raft organization and function in brush borders of epithelial cells. Mol Membr Biol.

[46] Parkin ET, Turner AJ, Hooper NM (2001). Differential effects of glycosphingolipids on the detergent-insolubility of the glycosylphosphatidylinositol-anchored membrane dipeptidase. The Biochemical journal.

[47] Mahfoud R, Manis A, Binnington B, Ackerley C, Lingwood CA (2010). A major fraction of glycosphingolipids in model and cellular cholesterol-containing membranes is undetectable by their binding proteins. The Journal of biological chemistry.

[48] Sonnino S (2009). Role of very long fatty acid-containing glycosphingolipids in membrane organization and cell signaling: the model of lactosylceramide in neutrophils. Glycoconj J.

[49] Sheikhpour M, Barani L, Kasaeian A (2017). Biomimetics in drug delivery systems: A critical review. Journal of controlled release: official journal of the Controlled Release Society.

[50] Allen TM, Cullis PR (2013). Liposomal drug delivery systems: from concept to clinical applications. Adv Drug Deliv Rev.

[51] Duncan GA, Bevan MA (2015). Computational design of nanoparticle drug delivery systems for selective targeting. Nanoscale.

[52] Tao A, Sinsermsuksakul P, Yang P (2006). Polyhedral silver nanocrystals with distinct scattering signatures. Angewandte Chemie.

[53] Wu HJ (2012). Membrane-protein binding measured with solution-phase plasmonic nanocube sensors. Nature methods.

[54] Gomez EW, Clack NG, Wu HJ, Groves JT (2009). Like-charge interactions between colloidal particles are asymmetric with respect to sign. Soft Matter.

[55] Wu HJ, Bevan MA (2005). Direct measurement of single and ensemble average particle-surface potential energy profiles. Langmuir.

[56] Wu HJ, Pangburn TO, Beckham RE, Bevan MA (2005). Measurement and interpretation of particle-particle and particle-wall interactions in levitated colloidal ensembles. Langmuir.

[57] Nayhouse M, Kwon JSI, Christofides PD, Orkoulas G (2013). Crystal shape modeling and control in protein crystal growth. Chemical Engineering Science.

[58] Fallahi-Sichani M, Linderman JJ (2009). Lipid raft-mediated regulation of G-protein coupled receptor signaling by ligands which influence receptor dimerization: a computational study. PLoS One.

