# Supplementary material for: Hetero-Multivalency of Pseudomonas aeruginosa Lectin LecA Binding to Model Membranes
Source: Sci Rep. 2018 May 30;8:8419. doi: 10.1038/s41598-018-26643-7 (PMC5976636; doi:10.1038/s41598-018-26643-7)
Supplement: Supplementary file 1 — Supplementary Information [file 41598_2018_26643_MOESM1_ESM.pdf]

Supplementary Information of

Hetero-Multivalency of *Pseudomonas aeruginosa* Lectin LecA Binding to Model Membranes

Authors: Nolan C. Worstell[1], Akshi Singla[1], Panatda Saenkham[2], Thushara Galbadage[2], Preeti Sule[2], Dongheon Lee[1], Alec Mohr[1], Joseph Sang-Il Kwon[1], Jeffrey D. Cirillo[2], and Hung-Jen Wu[1]*

Affiliations:
[1]Dept. of Chemical Engineering, Texas A&M University, College Station, Texas, USA

[2]Dept. of Microbial Pathogenesis and Immunology, Texas A&M Health Science Center, Bryan, Texas, USA

*corresponding author, email: hjwu@tamu.edu

Table of contents:

Materials and Methods S3

- Materials S3
- Nanocube Synthesis S3
- Vesicle Preparation S4
- Supported Lipid Bilayer Formation on Ag@SiO2 Nanocubes S4
- Supported Lipid Bilayer Formation on Silica Beads S4
- Nanocube Protein Binding Measurement S4
- Video Microscopy for Silica Particle Aggregation S5
- *P. aeruginosa* Liposomal Targeting S5
- Statistical Analysis and Regression S6
- Colloid Aggregation Kinetic Theory S6
- Kinetic Monte Carlo (kMC) Simulation S8

Supplementary text S1 ~ S3 S12

- Text S1: Measurement of LecA binding avidity using colloid aggregation kinetic theory S12
- Text S2: Positive cooperativity between Gb3 and other ligands S12
- Text S3: Binding cooperativity amongst moderate and weak ligands S13

Supplementary Figures S1 ~ S6 S14

- Fig S1: Calculated ϕ values at various [LecA] for a variety of bilayer mixtures S14
- Fig S2: Modeling LecA binding kinetics using the kMC simulation S15
- Fig S3: The schematic diagram for LecA- ligand binding kinetics S16

- Fig S4: Saturation curves for LecA binding to pure galactose terminated glycolipids given in semi-log form S17
- Fig S5: Saturation curves for LecA binding to Gb3, GM1, or AGM1 with LacCer bilayers as given in semi-log form S18
- Fig S6: Saturation curves for LecA binding Gb3, GM1, AGM1, or GalβCer mixed together given in semilog form S19

Supplementary Table S1 ~ S5 S20

- Table S1: Aggregation rate (k11) of Gb3, LacCer, Gb3/LacCer, and control bilayers S20
- Table S2: Binding cooperativity calculated using SI equation (2) S20
- Table S3: Nominal parameter values used in the kMC simulation S21
- Table S4: Hill's equation parameters obtained by fitting S21
- Table S5: Two-tailed Welch's t-tests for liposomal binding data S22

Supplementary Movies Description S23

Supplementary Data Files S23

Supplementary Reference S24

MATERIALS & METHODS

Materials

Ammonium hydroxide, bovine serum albumin Fraction V (BSA), copper (II) chloride dihydrate, ethanol, Pluronic F-127, polyvinylpyrrolidone (MW ~55,000) (PVP), tetraethyl orthosilicate (TEOS), silicone oil (useable range -50°C to +200°C) and tris-buffered saline (TBS) obtained as a 10x solution (1x working solution 20 mM Tris 0.9% NaCl pH ~7.4) were purchased from Sigma-Aldrich (St. Louis, Missouri). Silver(I) nitrate Premion® grade and the agar used for the LB agar plate, obtained as a powder, were purchased from Alfa-Aesar (Tewksbury, Massachusetts). 2-Propanol (iPA) and Texas Red™ 1,2-dihexadecanoyl-sn-glycero-3-phosphoethanolamine, triethylammonium salt (TR-DHPE) was purchased from Fisher Scientific (Pittsburgh, Pennsylvania). 1,5-Pentanediol (PD) was purchased from Acros Organics (Geel, Belgium). PA-IL from *Pseudomonas aeruginosa* (also known as LecA) was purchased from Elicityl (Crolles, France). 5.04 µm silica beads were purchased from Bangs Laboratories, Inc. (Fishers, Indiana). Calcium chloride was from BDH VWR Analytical (Radnor, Pennsylvania). The TBS solution used in bacterial binding was made using Tris from Research Products International, Corp. (Mt. Prospect, Illinois). The NaCl used to make the bacterial binding TBS solution along with the powder for Luria-Bertani (LB) broth were from Amresco (Solon, Ohio). HCl, ACS guaranteed reagent, for the bacterial binding TBS solution was obtained from EMD (Billerica, Massachusetts). Monosialogangliosides,GM1 (Galβ1-3GalNAcβ1-4(Neu5Acα2–3)Galβ1-4Glc-Ceramide), Gangliotetraosylceramide, AGM1,(Galβ1-3GalNAcβ1-4Galβ1-4Glc-Ceramide), Gangliotriosylceramide, AGM2, (GalNAcβ1-4Galβ1-4Glc-Ceramide), Globotriaosylceramide, Gb3, (Galα1-4Galß1-4Glc-Ceramide), Globotetrahexosylceramide, Gb4, (GalNAcß1-3Galα1-4Galß1-4Glc-Ceramide), Lactosylceramide, LacCer, (Galβ1-4Glc-Ceramide) and Galactosylceramide, GalβCer, (Galß-Ceramide) were purchased from Matreya, LLC. (State College, PA). 1-palmitoyl-2-oleoyl-sn-glycero-3-phosphocholine (POPC) and 1-palmitoyl-2-oleoyl-sn-glycero-3-phospho-L-serine (sodium salt) (POPS) were purchased from Avanti Polar Lipids (Alabaster, AL).

Nanocube Synthesis

The nanocube synthesis procedure is originally from Tao et al.[1] The silver nanocubes were synthesized via the polyol method which uses PVP as a structure-directing agent. In brief, the procedure was as follows. First, 0.2 g of $AgNO_3$ was dissolved into 10 mL of PD along with 30 µL of 82 mg/L $CuCl_2$ in PD. Next, 20 mL of PD was added to a 100 mL round bottom flask that was then heated to 130 °C with stirring in a 190°C silicon oil bath. After reaching 130 °C in the flask, 250 µL of the $AgNO_3$ solution along with 500 µL of a 20 g/L PVP in PD solution was added to the flask followed by a second addition of 500 µL from both the $AgNO_3$ and PVP solutions 35 seconds later. Then every following minute, 500 µL of each solution was added to the reactor until the solution turned a deep red color, about 15 minutes. After achieving a deep red color, the reaction was then allowed to cool and was washed by centrifugation using 200 proof ethanol.

The silica coating procedure was originally described in Wu et al.[2] and modified by Worstell et al.[3] First, 20 mL of the silver nanocube solution was washed into iPA via centrifugation and then added to a 250 mL round bottom flask along with 55 mL of iPA, 22.1 mL of MilliQ® water, 6.8 mL of TEOS, and 3.4 mL of 0.84% ammonium hydroxide. Next, the mixture was stirred at room temperature for 60 minutes before 50 mL of ethanol was added to stop the reaction. After stopping the reaction, the silica coated cubes were centrifuged and reconstituted

in 75 mL of iPA. The solution was then returned to the round bottom flask along with 22.1 mL of MilliQ® water, and 6.8 mL of TEOS. This solution was incubated at 60 °C for 10 hours before being washed with MilliQ® water. The silica coated nanocubes were stored in MilliQ® water at room temperature until use.

Vesicle Preparation

Small unilamellar vesicles (SUVs) were prepared via extrusion.[3] The procedure, in brief, is as follows. First, the desired compositions of lipids in chloroform solutions, prepared as per manufacturers recommendations, were mixed in a 25 mL round bottom flask and, then, dried using a rotary evaporator (Heidolph Hei-VAP Value®). Next, the dried lipids were reconstituted using MilliQ® water and extruded through a 100 nm polycarbonate filter (Whatman®) using a mini-extruder (Avanti Polar Lipids) resulting in a 3 g/L SUV solution.

Supported Lipid Bilayer Formation on Ag@SiO2 Nanocubes

Supported lipid bilayers were formed on the nanocubes using a modified vesicle fusion method.[3] 100 µL of the 3 g/L SUV solution was added to a 0.5 mL Eppendorf ® tube and vortex mixed for 20 seconds. Then, 10 µL of a concentrated nanocube solution was added to the tube and the tube was vortex mixed for 1 second. Following this, 110 µL of 2x TBS was added to the tube and vortex mixed for one second. These last two steps were repeated pipetting 10 µL of concentrated nanocube solution and 10 µL of 2x TBS each time until 100 µL of the nanocube solution was consumed. Then, the tube was vortex mixed for an additional 10 seconds and diluted with 1x TBS with 100 µM $CaCl_2$ to the desired nanocube concentration.

Supported Lipid Bilayer Formation on Silica Beads

Supported lipid bilayers were formed on the silica beads using a vesicle fusion method.[4] Initially, the stock silica bead solution was sonicated for 10 minutes and 5 µL of the solution was pipetted into a 0.5 mL Eppendorf ® tube. The beads were then washed three times with MilliQ® water, and the beads were resuspended in a final volume of 25 µL. In a separate 0.5 mL Eppendorf® tube, 25 µL of the SUV solution was added to 50 µL of 1x TBS and vortex mixed. Then, 25 µL of the washed beads were added to the SUV buffer solution and vortex mixed for 10 seconds and incubated for 10 minutes. Then, the beads were blocked with BSA for 1 hour followed by washing with 1x TBS resulting in a final volume of 100 µL of bilayer coated beads.

Nanocube Protein Binding Measurement

Bilayer coated nanocubes were incubated for 1 hour with 31.3 µL of 0.5 g/L BSA per 1250 µL of nanocube solution to reduce nonspecific binding. Then, the desired amount of LecA was added. For these experiments, 10 mol% POPS/90 mol% POPC lipid bilayer was used as a control. After addition of LecA, the test, control, and blank solutions were vortex mixed for 10 seconds each and pipetted as 20 µL aliquots into wells of a 384 well plate, 8 wells for the test, 4 wells for the control, and 4 wells for the blank solutions for each LecA concentration tested. Finally, the plate was read using a UV/Vis microplate reader spectrophotometer equipped with a CCD (FLUOstar Omega®, BMG-Labtech) to collect the extinction spectra every 13.3 minutes for a total of 80 minutes at room temperature. The resulting spectra were the results of averaging 200 flashes per well at a 1 nm resolution. The location of the quadrupole LSPR (Localized Surface Plasmon Resonance) peak (LSPR peak) was determined by 5th order polynomial fitting. The resulting LSPR peak shift was calculated from the average LSPR peak location of the 8 wells and then subtracted by the LSPR shift of the control lipid bilayer to give the total LSPR shift. It is worth noting that in contrast to single-molecule imaging technique, the solution phase

nanocube sensors measure the ensemble average of LecA binding events by collecting averaged binding profiles from nearly a million of nanocubes in the solution.[2] This nullifies the effect of variation in LecA distribution over nanocubes.

The saturation binding curves were fit by the Hill-Waud binding model[5]

$$\Delta\lambda_{LSPR} = \frac{V_m[LecA]^n}{K_h^n + [LecA]^n} \quad \textbf{(1)}$$

where K_h is the Hill's equation apparent dissociation constant, n is the Hill cooperativity coefficient, [LecA] is the concentration of LecA, and V_m is the maximum $\Delta\lambda_{LSPR}$ of the fully bound state. $\Delta\lambda_{LSPR}$ is the observed LSPR peak shift, which corresponds to the attachment of LecA on the lipid bilayer surface. To quantify the cooperative binding effect, we modified the heterogeneous cooperativity defined in our recent paper[6]:

$$\text{heterogeneous cooperativity } (\phi) = \Delta\lambda_{mix} - \sum_i \Delta\lambda_{pure,i} \quad \textbf{(2)}$$

where $\Delta\lambda_{mix}$ is the LSPR shift when LecA binds to a bilayer containing two different glycolipids, and $\Delta\lambda_{pure,i}$ is the LSPR shift when LecA binds to a bilayer containing the correspondent individual glycolipid, i. If no enhancement is observed between two different glycolipids, the ϕ value should be approximately zero. A positive (or negative) ϕ value indicates positive (or negative) cooperativity.

Video Microscopy for Silica Particle Aggregation

Wells of a 96 well-plate (Costar® 3370) were coated with polyethylene glycol (PEG) using Pluronic F-127. Initially, each well was rinsed four times by ethanol, and 250 µL ethanol was left to incubate in the wells for 30 minutes. Then, the wells were rinsed extensively with ethanol followed by five successive rinses of MilliQ® water. Following the cleaning steps, 250 µL of 5 g/L F-127 was added to each of the wells and left overnight. The next day, each well was rinsed five times with 250 µL of 1x TBS with 100 µM $CaCl_2$ while ensuring that none of the wells dried out when removing the solution. After the final rinse, the volume of solution left in each PEG-coated well was 96 µL of 1x TBS with 100 µM $CaCl_2$.

The procedure for video microscopy was adapted from Duncan et al.[7] 5 µL of 0.0324 g/L LecA solution in 1x TBS with 100µM $CaCl_2$ was added to a PEG-coated well and allowed to mix for at least 5 minutes. After LecA addition, 4 µL of the bilayer coated silica beads were added to the well and images were collected for 1.5 hours at an average frame rate of 12.8 frames/min. Imaging was performed using an inverted optical microscope (Axiovert® 200M, Carl Zeiss, Germany) with a 20x objective (Plan-NeoFluar, NA is 0.5, Carl Zeiss). Images were collected with a 14 bit CCD Camera (AxioCam HRc®, Carl Zeiss) operated in binning mode 1 (pixel size is 308nm/pixel, and image area is 1388 X 1040 $pixels^2$ = 427 X 320 $µm^2$). An image analysis algorithm coded in Matlab 2016a® was used to locate and track centers of each particle.[8,9] Particles were considered associated when the distance between particles centers was less than or equal to 2a+2 pixels (a is the particle radius).

P. aeruginosa Liposomal Targeting

Four kinds of fluorescent liposomes were prepared, i) 99 mol% POPC /1 mol% TR-DHPE, ii) 89 mol% POPC/10 mol% Gb3/1 mol% TR-DHPE, iii) 89 mol% POPC/10 mol% LacCer/1 mol% TR-DHPE and iv) 89 mol% POPC/5 mol% Gb3/5 mol% LacCer/1 mol% TR-DHPE. Lipids stored in organic solvents (chloroform for POPC or a chloroform/methanol/water mixture for glycolipids) were mixed to obtain the desired final composition. They were then dried using a

rotary evaporator (Heidolph Hei-VAP Value®), followed by rehydration with Milli-Q® water. SUVs were prepared by the standard extrusion protocol described in our prior publications.[3,6] The filters used for extrusion were Whatman® Track-Etched Nucleopore™ membrane having 19 mm diameter and 100 nm pore size.

P. aeruginosa strains PAO1/pJDC233 and Xen41 were cultured overnight in 3 ml LB medium at 37°C with shaking at 200 rpm and grown to an OD_{600} = 1.0. Cells were diluted 100 fold in LB, and 100 µl of this was added into 96 well plates (Greiner Bio-One µClear® product number 655096) and incubated at 37°C without shaking for 48 hours. Planktonic cells were carefully pipetted out, and attached cells were washed twice with TBS buffer (50 mM Tris-HCl, pH 7.6, 150 mM NaCl). After the washes, 100 µl of TBS buffer with 100 µM $CaCl_2$ containing Gb3, POPC, LacCer or Gb3/LacCer liposomes at different concentrations (0.3, 0.15, 0.0725 and 0 g/L) was added into 96 well plates and incubated at 37°C for 2 hours to facilitate liposome binding to bacterial cell membranes. Gentle rinsing with TBS buffer, twice, washed unbound liposomes away and bacterial cells were re-suspended in 100 µl of TBS with 100 µM $CaCl_2$ and mixed by through pipetting. The fluorescent signals of the liposome bound bacteria were detected using fluorescent spectrophotometer (EnVision™ 2104 Multilabel Reader, PerkinElmer®) at an Excitation/Emission wavelength of 580nm/620nm, respectively. Bacterial enumeration was performed by using 10-fold serial dilutions and plating on solid media (LB agar plate made from LB broth and 1.5% agar) to establish bacterial cell count (CFU/mL). The bacterial-liposome binding was represented as fluorescence signal per total number of bacteria. Each experiment was done in triplicate and the average value and standard error are reported.

Statistical Analysis and Regression

The data comprising each binding curve is given as a mean ± standard deviation (S.D.) where n = 8. The Hill-Waud model was then fit to the data for each binding curve via the Levenberg Marquardt algorithm in OriginPro 9.1® (OriginLab). This returned the calculated value, standard error, and R^2 value as well as the residuals, studentized residuals, and studentized deleted residuals. The parameter values and standard errors were reported in SI Table 4.

The *P. aeruginosa* liposomal binding data sets were tested for normality using the Kolmogrov-Smirnov test in OriginPro 9.1®. In all cases, we could not reject the null hypothesis that the data came from normal distributions. Therefore, it was reasonable to apply Welch's unequal variances t-test to the data. The results of Welch's t-test are graphically shown in Fig. 6 and the exact p-values and degrees of freedom are given in SI Table 5.

Colloid Aggregation Kinetic Theory

Duncan and Bevan measured Concanavalin A (ConA) binding to dextran by analyzing the aggregation kinetics of dextran-coated silica particles.[7] Here, we adapted the same analysis to monitor interactions between LecA and glycolipids. Compared to the nanocube protein binding measurement, this aggregation kinetic analysis allowed us to directly observe the energetic differences of LecA binding to different glycolipid mixtures. The analysis was conducted by measuring the rate of single particle disappearance using video microscopy.[7] The aggregation rate at short time scales was defined as:

$$\theta_1^{-1} = \frac{\phi_{1,0}}{\phi_1} = 1 + 4k_{11}\phi_{1,0}t \quad \textbf{(3)}$$

where t is time in seconds, and θ_1 is the fraction of single particles remaining over time. θ_1^{-1} was determined by taking the ratio of the number density of single particles at a certain time (ϕ_1) and at time = 0 ($\phi_{1,0}$). k_{11} is the rate constant for the self-aggregation of single particles.

We obtained k_{11} by fitting θ_1^{-1} vs. time data to equation (1). This aggregation kinetic model is valid for short time scales; thus, we fit the experimental data between $t = 0$ and $t = \tau$.[7] The definition of diffusion time (τ) is described in the following section. k_{11} is only a function of the pair potential between two particles, u_{pp}. As $k_{11} \gg 0$, it indicates strong binding between LecA and glycolipids. For weak interactions, $k_{11} \to 0$. By comparing k_{11} values, we can observe the energetic differences of LecA binding to different glycolipid mixtures.

In addition, we needed to identify the relevant time scales in which single particle-single particle interactions should dominate. This was done by calculating the mean diffusion time of first interaction for particles via the pair potential energy excluding biomolecular interactions in order to capture all relevant dynamics. The derivation of the theoretically predicted time scale based on the initial particle distribution is as follows.

Particle-Particle & Particle-Wall Interaction Potentials: For macromolecule-coated, micron-sized colloids, the interaction potential, u_{pw}, between a particle and a wall (i.e. well plate bottom) is given by Duncan et al. as[7]:

$$u_{pw}(h) = u_G(h) + u_v(h) + u_s(h) \quad \textbf{(4)}$$

where h is the particle-wall hard surface separation, and the subscripts are G (for gravity), v (for van der Waals), and S (for steric). The potential energy due to gravity is:

$$u_G(h) = \frac{4}{3}\pi a^3(\rho_p - \rho_f)gh \quad \textbf{(5)}$$

where a is the particle radius (2520 nm), g is the acceleration due to gravity, and ρ_p and ρ_f are the particle and fluid densities, respectively. [10] The van der Waals potential between half spaces was calculated using Lifshitz theory with consideration of retarded and screened interaction in dielectric media reported in our prior publication. [8] The values of water, polystyrene, and silica dielectric spectra are the same as those reported previously.[11] The particle-wall interactions were corrected by Derjaguin approximations.[12] For convenience, the van der Waals interactions calculated by Lifshitz theory and Derjaguin approximation are fitted to inverse power laws as:

$$u^{pw}{}_{vdW}(h) = -A_{pw}ah^{-p} \quad \textbf{(6)}$$

where A_{pw} and p for (6) are effective Hamaker constants. The fitted parameters for Equation (6) (silica particle-polystyrene well plate bottom) are A_{pw} = 0.3322 and p = 1.141. The steric potential represents the nonspecific osmotic repulsion due to macromolecules and is modeled as short range exponentials by Everett et al [10] as:

$$u_s^{pw}(h) = Be^{[-\kappa(h-\delta_{HW})]} \quad \textbf{(7)}$$

where κ is the inverse decay length and B is the intercept at separation. δ_{HW} is defined as the thickness of macromolecule layer (lipid bilayer is approximately 5 nm[10,13] and the thickness of F127 on polystyrene is 10 nm).[14] Thus, δ_{HW} = 16 nm).

Hydrodynamic Effects on Particle Diffusion: Particle diffusion in the bulk fluid is given by the Stokes-Einstein equation

$$D_o = \frac{k_B T}{6\pi\mu a} \quad \textbf{(8)}$$

When a particle stays near a planar surface, the particle-surface hydrodynamic interactions hinder the particle's diffusion parallel to the surface as given by:

$$D_{pw,\parallel}(h) = D_o f_{pw,\parallel}(h) \quad \textbf{(9)}$$

Where the correction factor, $f_{pw,\parallel}$ is the function of particle-surface separation, reported by Wu et al. [15]:

$$f_{pw,\parallel}(h) = \frac{12420\alpha(h)^2+5654\alpha(h)+100}{12420\alpha(h)^2+12233\alpha(h)+431} \quad \textbf{(10)}$$

where $\alpha(h) = h/a$. Thus, the average lateral diffusion of particle depends on the interactions between a particle and a planar surface as given by[16]

$$\langle D_{pw,\parallel}\rangle = \frac{\int D_{pw,\parallel}(h)p(h)dh}{\int p(h)dh} \quad \textbf{(11)}$$

where $p(h)$ represents the distribution of particle elevation that is determined by Boltzmann equation:

$$p(h) = Ae^{\frac{-u_{pw}}{k_BT}} \quad \textbf{(12)}$$

Besides the wall hindering diffusion, particle-particle multibody hydrodynamics also play a role in further hindering the lateral diffusion in concentrated colloidal systems. This diffusion along the line of particle centers is given as[7]:

$$D_{pp,\perp}(r) = \langle D_{pw,\parallel}\rangle f_{pp,\perp}(r) \quad \textbf{(13)}$$

where r is the particle center-particle center separation and $f_{pp,\perp}(r)$ is given by Honig et al.[17] as $\frac{1}{\beta(u)}$. β(u) is defined as:

$$\beta(u) \cong \frac{6u^2+13u+2}{6u^2+4u} \quad \textbf{(14)}$$

where $u = (r - 2a)/a$.

Finally, τ is the two dimensional diffusion time required for the particles to come into contact and defined as [18]:

$$\tau = \left(2\pi\phi_{1,0}D_{pp,\perp}(r)\right)^{-1} \ln\left[\frac{(\pi\phi_{1,0})^{-\frac{1}{2}}}{r_c}\right] \quad \textbf{(15)}$$

where r_c is the collision radius taken as r_c=a + 0.5 * $\delta_{HW,pp}$ where $\delta_{HW,pp}$=12 nm and the diffusion distance, $b = \left(\phi_{1,0}\right)^{-\frac{1}{2}} - 2a$ corresponding to the average separation between particles. τ is the value that we used as a cut off time for measuring single particle aggregation rates.

Kinetic Monte Carlo (kMC) Simulation

The kMC algorithm was implemented to model the kinetics of LecA binding to a membrane containing both high-affinity and low-affinity ligands.[19,20] The surface of lipid bilayer is modelled as a 250-by-250 square lattice sites (i.e. 212x212 nm^2) with a periodic boundary condition, and ligands are randomly distributed on the surface. The details of the kMC simulation are described below.

Microscopic Phenomena of kMC: The LecA-ligand binding kinetics are described by five microscopic phenomena as follows:

- Ligands and LecA-ligand complexes on a lipid bilayer surface migrate due to the fluidity of the lipid bilayer
- LecA proteins diffuse within the solution.
- If a LecA is sufficiently close to a ligand on the surface, LecA can attach to the surface by binding to the ligand.
- A membrane-bound LecA binds to an additional ligand if the bound LecA has an unfilled binding site and a ligand is sufficiently close to the LecA.
- A ligand can dissociate from a membrane-bound LecA, and the LecA will detach from the lipid bilayer after all ligands dissociate from it.

For the purpose of this study, the above descriptions are simplified with the following two assumptions. First, the simulation domain is restricted to two-dimensions. To this end, the transport of LecA proteins to and from the surface via diffusion is described by effective association and dissociation rate constants from the literature. Second, because the diffusivity of membrane-bound lectins is almost two orders of magnitude lower than the glycolipid ligand,[21] we assume LecA-ligand complexes on the surface are immobile.

Surface kinetics: As a LecA molecule has two binding sites facing a membrane surface, the LecA molecule will bind to or dissociate from ligands in a stepwise manner, which results in 12 reactions to be considered in the kMC simulation (SI Figure 3). The steps from solution-phase LecA to membrane-bound LecA were treated by the effective rate constants $\mathrm{k_{f,H}}$, $\mathrm{k_{f,L}}$, $\mathrm{k_{r,H}}$, and $\mathrm{k_{r,L}}$ (the second subscripts, H and L, represent the rate constant corresponding to high-affinity and low affinity ligands). First, the attachment and detachment rates are defined as:

$$\mathrm{r_{a,H}} = 2\mathrm{k_{f,H}CR}_{H}, \qquad \mathrm{r_{a,L}} = 2\mathrm{k_{f,L}CR}_{L}, \qquad \mathrm{r_{d,H}} = \mathrm{k_{r,H}B_{1,0}}, \qquad \mathrm{r_{d,L}} = \mathrm{k_{r,L}B_{0,1}} \tag{16}$$

where $\mathrm{r_a}$ is the attachment rate from solution to the surface, C is the LecA concentration in solution, $\mathrm{r_d}$ is the LecA detachment rate from the membrane to solution, R is the number of ligand, and $\mathrm{B_{i,j}}$ is the number of LecA binding to i and j number of high-affinity and low-affinity ligands. Here, a factor of two is multiplied because a LecA protein is symmetric molecule with two identical binding sites.

Since a LecA can take up to two ligands in a membrane, a membrane-bound LecA can associate with or dissociate from additional ligands, which are termed as forward and backward reactions, respectively, hereafter (SI Figure 3). The reaction rates of these surface binding events on the membrane are computed as follows

$$\begin{gathered}\mathrm{r_{1,H}} = \mathrm{k_{1,H}B_{1,0}R}_{H}, \quad \mathrm{r_{1,L}} = \mathrm{k_{2,L}B_{0,1}R}_{L}, \quad r_{-1,H} = 2\mathrm{k_{-1,H}B_{2,0}}, \quad r_{-1,L} = 2\mathrm{k_{-1,L}B_{0,2}} \\ \mathrm{r_{2,H}} = \mathrm{k_{1,H}B_{0,1}R}_{H}, \quad \mathrm{r_{2,L}} = \mathrm{k_{1,L}B_{1,0}R}_{H}, \quad r_{-2,H} = \mathrm{k_{-1,H}B_{1,1}}, \quad r_{-2,L} = \mathrm{k_{-1,L}B_{1,1}}\end{gathered} \tag{17}$$

where $\mathrm{k_1}$ and $\mathrm{k_{-1}}$ are the forward and backward reaction rate on membrane surface.

Finally, the ligand migration rate is defined as[19,20]

$$\mathrm{r_{m,k}} = \mathrm{R_k}\frac{\mathrm{k_{m,k}}}{\mathrm{l}^2}, \forall\, k \in \{\text{'H'}, \text{'L'}\} \qquad \textbf{(18)}$$

where $\mathrm{k_{m,k}}$ is the migration rate constant of ligand k, and l is the distance between two lattice sites.

Kinetic Monte Carlo Implementation: An event is selected based on a random number and the total reaction rate, r_t, which is defined as

$$r_t = \sum_{k\in H,L} r_{a,k} + r_{d,k} + r_{-2,k} + r_{-1,k} + r_{1,k} + r_{2,k} + r_{m,k} \qquad \textbf{(19)}$$

In order to execute an event, a uniform random number, $\xi_1 \in [0,1)$, is sampled. If $\xi_1 \le r_{a,L}/r_t$, the attachment event with low-affinity ligand is selected. If $r_{a,L}/r_t < \xi_1 \le (r_{a,L} + r_{d,L})/r_t$, the detachment event with low affinity ligand is selected. If $(r_{a,L} + r_{d,L})/r_t < \xi_1 \le (r_{a,L} + r_{d,L} + r_{1,L})/r_t$, the forward reaction from $B_{0,1}$ to $B_{0,2}$ is selected. If $(r_{a,L} + r_{d,L} + r_{1,L})/r_t < \xi_1 \le (r_{a,L} + r_{d,L} + r_{1,L} + r_{-1,L})$, the backward reaction from $B_{0,2}$ to $B_{0,1}$ is selected to occur; if , $(r_{a,L} + r_{d,L} + r_{1,L} + r_{-1,L})/r_t < \xi_1 \le (r_{a,L} + r_{d,L} + r_{1,L} + r_{-1,L} + r_{-2,L})$, the backward reaction from $B_{1,1}$ to $B_{0,1}$ is selected; if $(r_{a,L} + r_{d,L} + r_{1,L} + r_{-1,L} + r_{-2,L})/r_t < \xi_1 \le (r_{a,L} + r_{d,L} + r_{1,L} + r_{-1,L} + r_{-2,L} + r_{2,L})/r_t$, the forward reaction from $B_{1,0}$ to $B_{1,1}$ is selected; if $(r_{a,L} + r_{d,L} + r_{1,L} + r_{-1,L} + r_{-2,L} + r_{2,L})/r_t < \xi_1 \le (r_{a,L} + r_{d,L} + r_{1,L} + r_{-1,L} + r_{-2,L} + r_{2,L} + r_{m,L})/r_t$, the migration event of lower affinity ligand is selected. Inequalities for selecting events related to high-affinity ligands can be written similarly, which are not shown here.

When an attachment event ($\mathrm{r_{a,k}}$) is selected, a free ligand of type k is randomly selected to associate with an incoming LecA protein. After a ligand for binding is selected, it is required to check whether there is enough free space around the selected ligand for the LecA molecule without overlapping with other LecA molecules that are already bound to the host cell membrane. If there is not enough space for an incoming LecA molecule, the attachment will be rejected. As the membrane becomes more crowded with an increasing number of membrane-bound LecA molecules, the available space for an additional LecA to attach to the host cell membrane decreases significantly; hence, the rejection rate will increase accordingly.

When a detachment event ($\mathrm{r}_{d,\mathrm{k}}$) is selected, one LecA molecule bound to one ligand of type k is randomly selected and the LecA molecule dissociates from the ligand. Whenever attachment or detachment events occur, the concentration of LecA in solution is updated via a mass balance by counting the number of proteins undergoing attachment and detachment processes.

When a forward reaction event on the membrane surface is selected, a free ligand of the corresponding type will bind to a LecA molecule attached to a ligand. Here, it is required to check whether there are any free ligands sufficiently close to the selected binding site, which is determined by the distance between the binding site and free ligands on the membrane. If the distance is smaller than the threshold distance (l_c), the corresponding ligand is classified as a free ligand that can bind with the LecA molecule. If there are no ligands close to the selected binding site, the forward reaction will not occur; if there is more than one available ligand, a ligand is randomly selected for the binding event.

Similarly, when a backward reaction event occurs, one bound LecA molecule is randomly selected, and one of its bound ligands is randomly chosen for dissociation.

When a migration event ($r_{m,k}$) happens, one free ligand of type k is randomly selected and moves to one of its neighbouring empty sites.

After one event is selected and proceeds as described above, the time increment for the selected event is calculated by generating a new random number $\xi_2 \in [0,1)$, and the time increment is computed as follows[22]

$$\tau = -\frac{\ln \xi_2}{r_t} \quad \textbf{(20)}$$

and the simulation will proceed by $t + \tau$ seconds. The kMC simulation is written in C#, and 50 trials are computed to calculate the average kinetics.

Parameter Selection: The distance between two lattice sites (l) is 0.85nm, which is equivalent to the head group size of DOPC in bilayer ($\sqrt{0.72nm^2} = 0.85nm$).[23] Because the size of a LecA subunit is ~2nm, we used 3nm for the value of the threshold radius (l_c). The nominal parameters for the high-affinity and low affinity ligands are listed in SI Table 3. The migration constant ($k_{m,k}$) of ligands was estimated by the average DOPC lipid diffusivity ($8.25 \times 10^{-12}\ m^2/s$).[23] The kinetic constants of LecA are not available. Lauer et al. analyzed the binding kinetics of cholera toxin subunit B (CTB) using the stepwise binding model, allowing us to estimate the kinetic constants. For the high-affinity ligand, k_f and k_r were acquired from the fundamental forward and reverse rate constants reported by Lauer et al. (k_1 and k_{-1} in the reference[24]). Because the dissociation constants are associated with releasing the binding between LecA and its ligands, we assume $k_r = k_{-1}$. k_1 is the surface forward rate constant without the contribution of reactants' surface diffusion; thus, we cannot use the fitted surface rate constant reported by Lauer et al. Instead, we used the parameter estimated by the interaction of membrane-bound antibody-antigen complexes reported by Sengers et al.[25] Because the equilibrium dissociation constant of the antibody-antigen system is an order of magnitude lower than LecA binding system, we chose $k_1 = 0.07\ \mu m^2 s^{-1} molecule^{-1}$, instead of the value ($0.7\ \mu m^2 s^{-1} molecule^{-1}$) reported by Sengers et al.[25] We reduced the forward rate constants (k_f and k_1) 100-, 300-, and 1000-fold for the low-affinity ligands. The other rate constants of the low-affinity ligand remained same as the high-affinity ligands. It is worth noting that we have varied the rate constants two-orders of magnitude higher and lower to observe the influence of parameter selection. The results indicated that the qualitative phenomenon of hetero-multivalency remains the same as what was described in the main text.

SUPPLEMENTARY TEXT:

Supplementary text S1

Measurement of LecA binding avidity using colloid aggregation kinetic theory: We used a video microscopy technique developed by Duncan and Bevan to directly measure the binding avidity.[7] This video microscopy technique not only offers us a measurement of LecA binding avidity but also allows for direct visualization of LecA-glycolipid ligand interactions.

To implement this technique, we first deposited lipid bilayers containing the desired glycolipid ligands onto 5 μm silica beads. Then, LecA was bound to glycolipid ligands thereby linking two silica particles. (Fig. 4) It is worth noting that the colloid aggregation assay measures the average potential energy between two particles, which is a function of LecA-glycolipid interaction as well as the average number of bound LecA presenting in the contact area between the two particles. To compare the binding energy between LecA and glycolipid, we have to limit the number of LecA molecules presenting in the junction of the two particles. Here, we conducted the aggregation experiment at a low LecA concentration (~30nM), which is correspondent to ~5% of the binding saturation (Fig.2b). Under this condition, only 1~2 LecA molecules should be present in the interaction area between two particles, if LecA molecules are uniformly distributed. Thus, the measured aggregation rate is primarily associated with the binding energy between LecA and glycolipid ligands.

Supplemental videos show the aggregation kinetics of lipid bilayer coated particles. If the binding avidity was strong (1 mol% Gb3), the aggregation occurred immediately after two particles touch (SI Movie 1). If the binding avidity is slightly weaker (1 mol% Gb3 + 4 mol% LacCer), some particles could collide and bounce apart (SI Movie 2). At very low binding avidity (4 mol% LacCer), the linkages between particles were not stable enough to maintain the particle clusters (SI Movie 3). This aggregation process can be quantified by measuring the rate of single particle disappearance. The calculated aggregation rate (k_{11}) significantly decreased when LacCer was mixed with Gb3 in the lipid bilayer, indicating the binding avidity reduced in the Gb3/LacCer mixture. (SI Table 1)

Supplementary text S2

Positive cooperativity between Gb3 and other ligands: Besides LacCer, we explored hetero-multivalent binding cooperativity between Gb3 and other weak glycolipid ligands. We first mixed Gb3 with the simplest glycolipid, galactosylceramide (GalβCer), which consists of a single β–galactose residue. GalβCer is highly abundant in the brain and intestinal epithelial cells[26,27]; thus, it may play a role in the LecA binding process. As expected, we observed positive cooperativity when 8 mol% GalβCer was mixed with 1 mol% Gb3 (Fig. 5a and SI Table 2). However, the degree of enhancement is lower than the Gb3/LacCer combination. According to the observations from the kMC simulation, this is probably because the binding affinity of GalβCer is weaker than LacCer.

LecA also has weak binding affinity to N-acetylgalactosamine (GalNAc) terminated glycans.[28-30] Thus, we also investigated the binding cooperativities of two GalNAc terminated glycolipids, Gb4, and AGM2 (Fig. 5b). In this case, we only conducted LecA binding experiments at 3 μM, which is the highest concentration in the previous binding curves. At 8 mol% of pure AGM2, no significant LecA binding was observed. The degree of LecA binding to 8 mol% pure Gb4 is similar to 8 mol% pure LacCer systems. When either AGM2 or Gb4 was mixed with 1 mol% Gb3, we again observed positive cooperativity between strong and weak

binding ligands. This shows that Gb3 could form a partnership with GalNAc terminated glycolipids, leading to positive cooperativity.

It is reasonable to assume the moderate ligands (AGM1 and GM1) could also be activated via the same RD mechanism. As expected in Fig. 5c, we observed that 1 mol% Gb3 ligands could activate either 1 mol% AGM1 or GM1, leading to higher LecA attachment. In addition to testing cooperativity between Gb3 and moderate ligands, we compared the cooperativity amongst the moderate ligands themselves. In the mixture of 1 mol% of GM1 and 1 mol% of AGM1, we observed much greater LecA attachment than the values in bilayers containing 1 mol% of either GM1 or AGM1. The increase of available ligands in the lipid bilayer is probably the reason for the increased cooperativity among moderate ligands.

Supplementary text S3

Binding cooperativity amongst moderate and weak ligands: In addition to the strong ligand (Gb3), we wondered if the moderate ligands (AGM1 and GM1) were sufficient to activate weak ligands, leading to higher LecA attachment. First, we investigated the binding cooperativity between LacCer and the moderate ligands (Fig. 5d). We observed positive cooperativity between 1 mol% of each moderate ligand, individually, with 4 or 8 mol% of LacCer. This observation indicated that the moderate ligands were able to activate LacCer. We also examined the change of cooperativity at different LecA concentrations (SI Fig. 1). Similar to Gb3/LacCer system, the cooperativity became significant when the LecA concentration reached a threshold value. However, the threshold concentration of the moderate ligands (~0.5 μM) was higher than the threshold of Gb3 (0.1μM). As discussed above, the threshold of LecA concentration is probably dominated by the first binding step, which is associated with the dissociation constant of the ligand with higher affinity. Thus, we observed cooperativity significantly increased after the LecA concentration reached the dissociation constants of the moderate ligands (~0.5 μM).

The cooperativity between the moderate ligands and GalβCer is not as significant as LacCer (Fig. 5e). The calculated heterogeneous cooperativity for GalβCer with GM1 is slightly higher than AGM1; however, the cooperative values for both GM1 and AGM1 systems are below than two standard deviations (SI Table 2). As a result, we were prevented from making any definitive statement about the positive cooperativity between GalβCer and the moderate ligands.

SUPPLEMENTARY FIGURES:

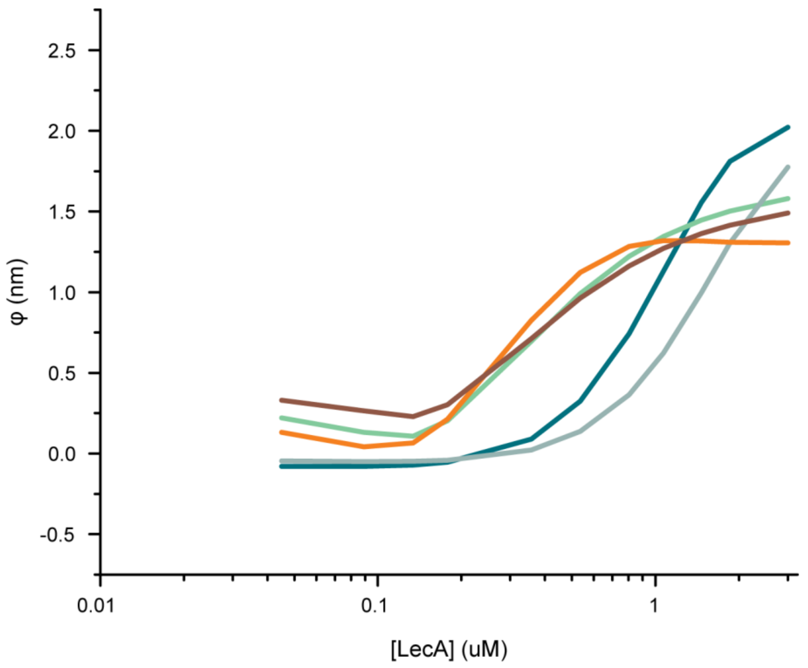

	Lipid Compositions (mol%)			
	Gb3	GM1	AGM1	LacCer
●	0	1	0	8
●	0	0	1	8
●	1	1	0	0
●	1	0	0	4
●	1	0	1	0

SI Figure 1: Calculated ϕ values at various [LecA] for a variety of bilayer mixtures.

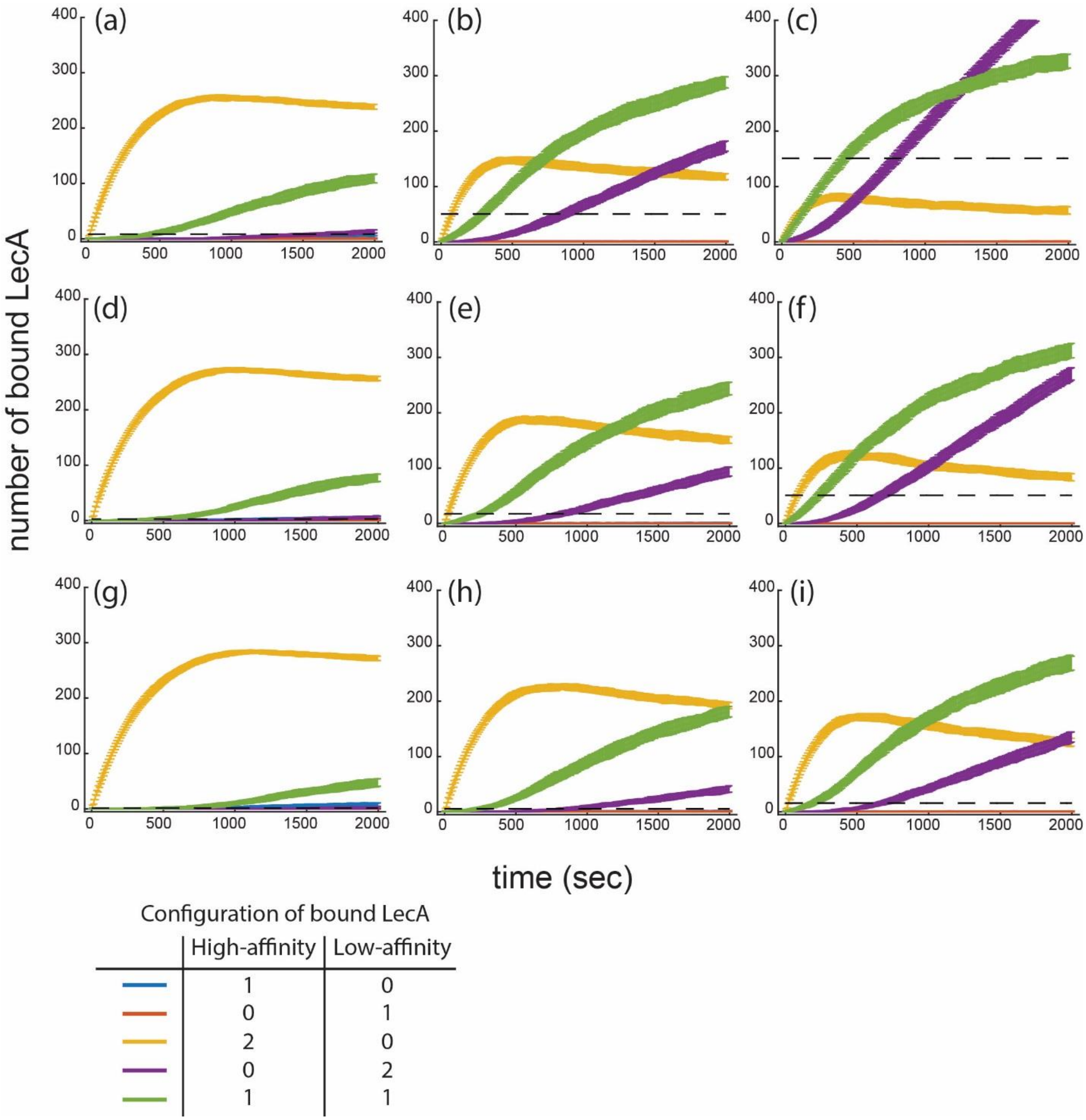

SI Figure 2: Modeling LecA binding kinetics using the kMC simulation. LecA binding to a membrane surface (250x250 sites) containing 1 mol% of high-affinity ligands and various densities of low-affinity ligand with different affinities: (a) 0.5 mol% ($\boldsymbol{K_{d,low} = 100K_{d,high}}$), (b) 3 mol% ($\boldsymbol{K_{d,low} = 100K_{d,high}}$), (c) 9 mol% ($\boldsymbol{K_{d,low} = 100K_{d,high}}$), (d) 0.5 mol% ($\boldsymbol{K_{d,low} = 300K_{d,high}}$), (e) 3 mol% ($\boldsymbol{K_{d,low} = 300K_{d,high}}$), (f) 9 mol% ($\boldsymbol{K_{d,low} = 300K_{d,high}}$), (g) 0.5 mol% ($\boldsymbol{K_{d,low} = 1000K_{d,high}}$), (h) 3 mol% ($\boldsymbol{K_{d,low} = 1000K_{d,high}}$), (i) 9 mol% ($\boldsymbol{K_{d,low} = 1000K_{d,high}}$). Each curve represents the number of bound LecA at different binding configurations shown in the figure legend. The dash line shows the maximum number of bound LecA at 2,000 s when the membrane contains the same density of low-affinity ligands without any high-affinity ligands. All data represented as average ± S.D from 50 kMC simulations.

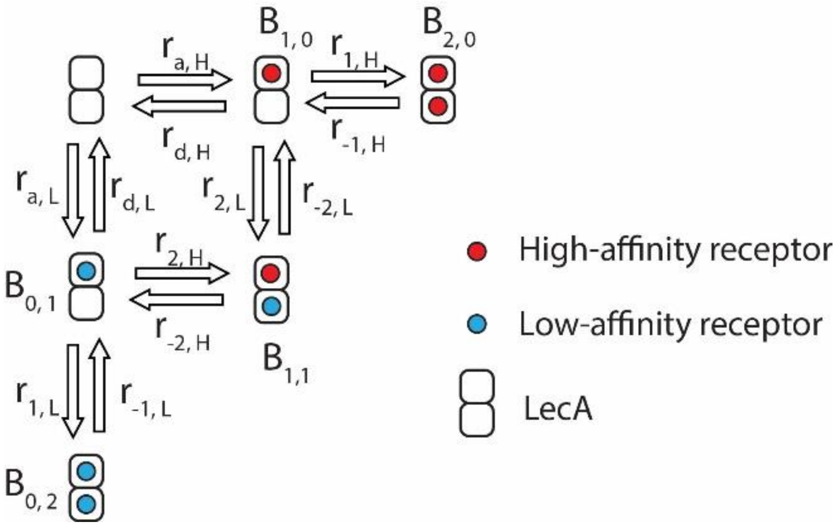

SI Figure 3: The schematic diagram for LecA- ligand binding kinetics. $B_{i,j}$ is a LecA bound to *i* and *j* number of high-affinity and low-affinity ligands, respectively

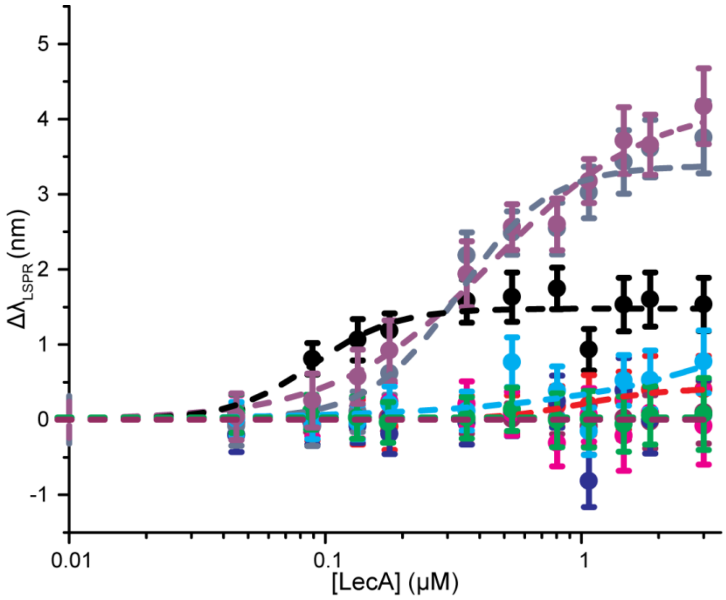

	Lipid Compositions (mol%)				
	Gb3	GM1	AGM1	LacCer	GalβCer
●	0	5	0	0	0
●	0	0	5	0	0
●	1	0	0	0	0
●	0	0	0	8	0
●	0	0	1	0	0
●	0	1	0	0	0
●	0	0	0	0	4
●	0	0	0	0	8
●	0	0	0	4	0

SI Figure 4: Saturation curves for LecA binding to pure galactose terminated glycolipids given in semi-log form. The data are represented as mean±S.D. (n=8). The dashed lines are the Hill equation fits to the data.

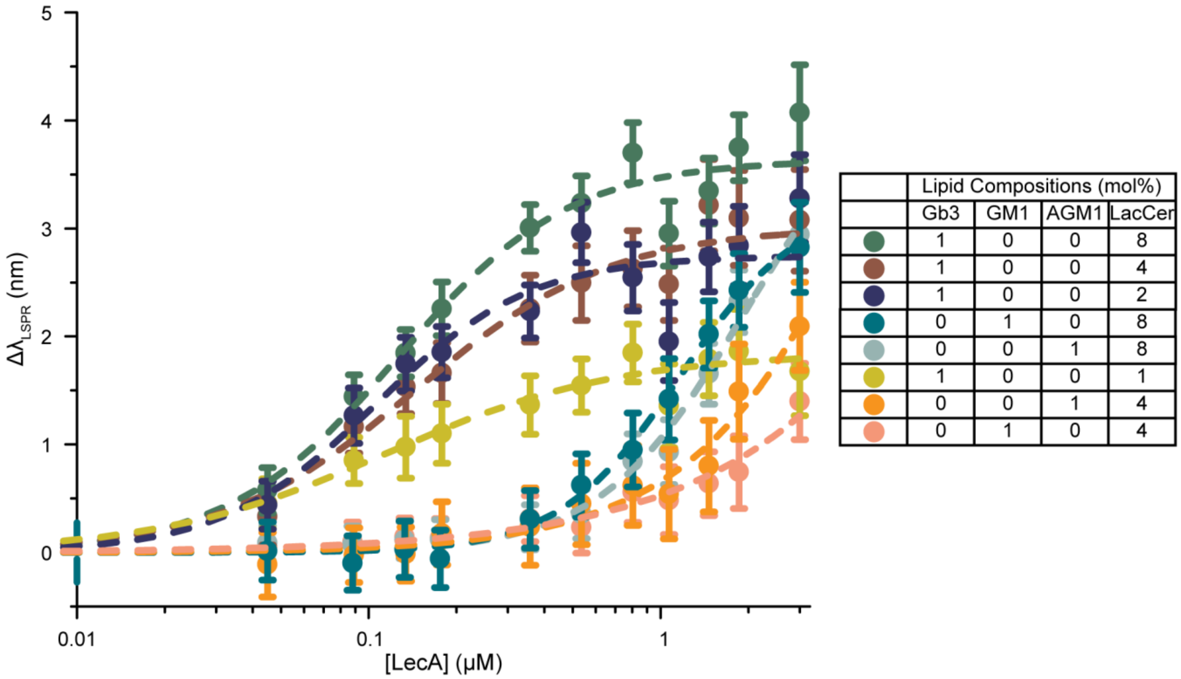

SI Figure 5: Saturation curves for LecA binding to Gb3, GM1, or AGM1 with LacCer bilayers as given in semi-log form. The data are represented as mean±S.D. (n=8). The dashed lines are the Hill equation fits to the data.

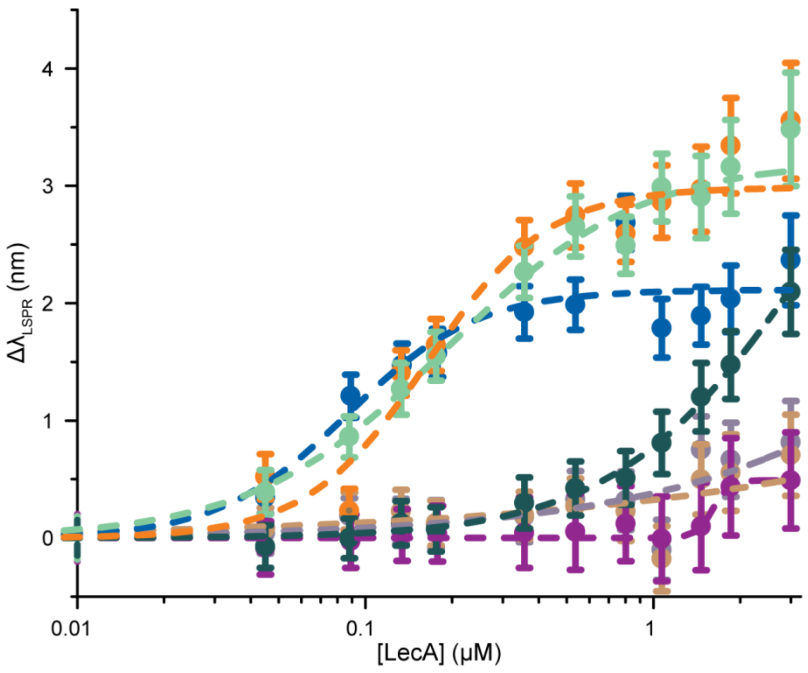

	Lipid Compositions (mol%)			
	GB3	GM1	AGM1	GalβCer
	1	1	0	0
	1	0	1	0
	1	0	0	8
	0	1	1	0
	0	0	1	8
	0	1	0	8
	0	0	1	8

SI Figure 6: Saturation curves for LecA binding Gb3, GM1, AGM1, or GalβCer mixed together given in semilog form. The data are represented as mean±S.D. (n=8). The dashed lines are the Hill equation fits to the data.

SUPPLEMENTARY TABLES:

SI Table 1: Aggregation rate (k_{11}) of Gb3, LacCer, Gb3/LacCer, and control bilayers. k_{11} is represented as mean ± S.D. (n =2). N/A indicates that the S.D. was not determined.

Lipid Compositions (mol %)				Fitted
Gb3	LacCer	POPS	POPC	k_{11} (μm^2/(bead*s))
1	0	10	89	0.20 ± 0.03
0	4	10	86	0.03 ± N/A
1	4	10	85	0.13 ± 0.01
0	0	10	90	0.01 ± N/A

SI Table 2: Binding cooperativity, ϕ, calculated using SI equation (2) with 3 μM LecA LSPR shifts. The values are the mean ± S.E. (n=8).

**Lipid compositions (mol%)**							
**Gb3**	**GM1**	**AGM1**	**LacCer**	**GalßCer**	**Gb4**	**AGM2**	**φ**
1	1	0	0	0	0	0	1.9 ± 0.3
1	0	1	0	0	0	0	1.6 ± 0.3
1	0	0	1	0	0	0	0.2 ± 0.2
1	0	0	2	0	0	0	1.8 ± 0.2
1	0	0	4	0	0	0	1.6 ± 0.2
1	0	0	8	0	0	0	1.8 ± 0.2
1	0	0	0	8	0	0	0.8 ± 0.2
1	0	0	0	0	8	0	0.5 ± 0.2
1	0	0	0	0	0	8	0.7 ± 0.2
0	1	1	0	0	0	0	1.6 ± 0.3
0	1	0	4	0	0	0	1.4 ± 0.2
0	1	0	8	0	0	0	2.0 ± 0.3
0	1	0	0	8	0	0	0.6 ± 0.2
0	0	1	4	0	0	0	1.7 ± 0.2
0	0	1	8	0	0	0	1.8 ± 0.2
0	0	1	0	4	0	0	0.2 ± 0.3
0	0	1	0	8	0	0	0.4 ± 0.2

SI Table 3: Nominal parameter values used in the kMC simulation

	High-affinity ligand	Low-affinity ligand (100-fold weaker)	Low-affinity ligand (300-fold weaker)	Low-affinity ligand (1,000-fold weaker)
$k_f\ (M^{-1} \cdot s^{-1})$	2.8×10^4	2.8×10^2	9.3×10^1	2.8×10^1
$k_r\ (s^{-1})$	3.2×10^{-3}	3.2×10^{-3}	3.2×10^{-3}	3.2×10^{-3}
$k_1\ (\mu m^2 s^{-1})$	7×10^{-2}	7×10^{-4}	2.3×10^{-4}	7×10^{-5}
$k_{-1}\ (s^{-1})$	3.2×10^{-3}	3.2×10^{-3}	3.2×10^{-3}	3.2×10^{-3}
$k_m\ (m^2/s)$	8.25×10^{-12}	8.25×10^{-12}	8.25×10^{-12}	8.25×10^{-12}

SI Table 4: Hill's equation parameters obtained by fitting in OriginLab. A * indicates that fitting was highly uncertain due to the data not reaching a plateau and – indicates fitting did not converge. The values are represented as a mean±SE (where the standard error of the fit is based on fitting through 96 points for each curve).

**Lipid Compositions (mol%)**							**Fitted Parameters**			
**Gb3**	**GM1**	**AGM1**	**LacCer**	**Galß**	**POPS**	**POPC**	**V_m (µM)**	**K_h (µM)**	**n**	
1	0	0	0	0	10	89	1.53 ± 0.02	0.09 ± 0.00	2.68 ± 0.24	
0	1	0	0	0	10	89	0.10 ± 0.03	0.13 ± 0.00	0.00 ± 0.40	*
0	5	0	0	0	10	85	4.37 ± 0.08	0.48 ± 0.02	1.36 ± 0.05	
0	0	1	0	0	10	89	0.44 ± 0.06	1.10 ± 0.17	2.64 ± 0.77	
0	0	5	0	0	10	85	3.58 ± 0.04	0.37 ± 0.01	2.11 ± 0.08	
0	0	0	4	0	10	86	-	-	-	*
0	0	0	8	0	10	82	$6.41*10^3 \pm 3.48*10^7$	$3.76*10^6 \pm 3.15*10^{10}$	0.65 ± 0.22	*
0	0	0	0	4	10	86	0.12 ± 0.04	0.18 ± 0.00	0.00 ± 0.42	*
0	0	0	0	8	10	82	-	-	-	*
1	1	0	0	0	10	88	3.24 ± 0.04	0.19 ± 0.01	1.26 ± 0.04	
1	0	1	0	0	10	88	3.30 ± 0.04	0.20 ± 0.01	1.50 ± 0.07	
1	0	0	1	0	10	88	1.83 ± 0.04	0.12 ± 0.01	1.08 ± 0.07	
1	0	0	2	0	10	87	2.81 ± 0.04	0.11 ± 0.01	1.39 ± 0.11	
1	0	0	4	0	10	85	3.18 ± 0.04	0.17 ± 0.01	1.16 ± 0.05	
1	0	0	8	0	10	81	3.75 ± 0.04	0.13 ± 0.00	1.35 ± 0.05	
1	0	0	0	8	10	81	2.11 ± 0.02	0.09 ± 0.00	1.92 ± 0.14	
0	1	1	0	0	10	88	4.24 ± 0.51	3.02 ± 0.51	1.34 ± 0.07	*
0	1	0	4	0	10	85	$9.30*10^3 \pm 1.96*10^7$	$3.81*10^5 \pm 1.05*10^9$	0.8 ± 0.1	*
0	1	0	8	0	10	81	3.26 ± 0.08	1.17 ± 0.03	2.08 ± 0.08	
0	1	0	0	8	10	81	$3.21*10^2 \pm 8.42*10^4$	$1.84*10^4 + 6.86*10^6$	0.71 ± 0.22	*
0	0	1	4	0	10	85	14.32 ± 16.95	13.67 ± 18.53	1.15 ± 0.13	*
0	0	1	8	0	10	81	3.91 ± 0.13	1.66 ± 0.07	2.00 ± 0.08	
0	0	1	0	4	10	85	0.49 ± 0.03	1.61 ± 0.05	14.62 ± 4.13	
0	0	1	0	8	10	81	$5.95*10^3 \pm 1.85*10^7$	$6.12*10^5 \pm 2.62*10^9$	0.73 ± 0.18	*

SI Table 5: Two-tailed Welch's t-tests for liposomal binding data given in Fig. 6.

Species	concentration 1 (g/L)	Liposome 1	concentration 2 (g/L)	Liposome 2	p value	Degrees of freedom
PAO1	0.3	Gb3	0.3	Gb3+LacCer	0.02215	3.98793
PAO1	0.3	Gb3	0.15	Gb3+LacCer	0.63684	2.1086
PAO1	0.15	Gb3	0.15	Gb3+LacCer	3.58E-05	3.98584
PAO1	0.0725	Gb3	0.0725	Gb3+LacCer	3.71E-04	3.30326
PAO1	0.3	POPC	0.3	Gb3	0.58411	2.465
PAO1	0.3	POPC	0.3	Gb3+LacCer	0.01745	2.51739
PAO1	0.3	POPC	0.15	Gb3+LacCer	0.79557	2.87554
PAO1	0.3	POPC	0.0725	Gb3+LacCer	0.00576	2.93179
PAO1	0.3	POPC	0.3	LacCer	0.07962	3.12156
PAO1	0.15	POPC	0.15	Gb3	0.78297	3.0836
PAO1	0.15	POPC	0.15	Gb3+LacCer	9.82E-04	2.97826
PAO1	0.15	POPC	0.0725	Gb3+LacCer	0.01402	3.03903
PAO1	0.15	POPC	0.15	LacCer	0.28177	2.03355
PAO1	0.0725	POPC	0.0725	Gb3	0.14898	3.58562
PAO1	0.0725	POPC	0.0725	Gb3+LacCer	1.39E-04	3.92161
PAO1	0.0725	POPC	0.0725	LacCer	0.00628	2.17085
Xen41	0.3	Gb3	0.3	Gb3+LacCer	0.0536	3.58036
Xen41	0.15	Gb3	0.15	Gb3+LacCer	0.02741	2.77626
Xen41	0.0725	Gb3	0.0725	Gb3+LacCer	0.02189	2.05894
Xen41	0.3	POPC	0.3	Gb3	0.02749	2.74512
Xen41	0.3	POPC	0.15	Gb3	0.31673	3.91445
Xen41	0.3	POPC	0.3	Gb3+LacCer	0.01478	2.37532
Xen41	0.3	POPC	0.15	Gb3+LacCer	0.04288	2.58663
Xen41	0.3	POPC	0.0725	Gb3+LacCer	0.58316	2.89825
Xen41	0.3	POPC	0.3	LacCer	0.7375	2.5455
Xen41	0.15	POPC	0.15	Gb3	0.0668	3.8223
Xen41	0.15	POPC	0.15	Gb3+LacCer	0.00953	3.14037
Xen41	0.15	POPC	0.15	LacCer	0.54893	2.18579
Xen41	0.0725	POPC	0.0725	Gb3	0.02294	3.39069
Xen41	0.0725	POPC	0.0725	Gb3+LacCer	0.0192	2.02385
Xen41	0.0725	POPC	0.0725	LacCer	0.08573	2.76924

SUPPLEMENTARY MOVIES:

SI Movie 1: The aggregation of silica particles coated with lipid bilayer containing 1 mol% Gb3. The time interval per frame is 4.8 s.

SI Movie 2: The aggregation of silica particles coated with lipid bilayer containing 1 mol% Gb3 + 4 mol% LacCer. The time interval per frame is 4.8 s.

SI Movie 3: The aggregation of silica particles coated with lipid bilayer containing 4 mol% LacCer. The time interval per frame is 4.8 s.

SUPPLEMENTARY DATA FILES:

In the kMC simulation, we varied the density of the low-affinity ligand from 0.5 mol% to 9 mol%. We have not reported all the simulation data in the Figure 3 and SI Figure 2 because the trend of hetero-multivalency effect is same over all the tested conditions. The following spreadsheet files contain the modeling data of the kMC simulation that were not shown in the figures.

SI Dataset 1: The simulation data of LecA binding kinetics when the affinity of the weak ligand is 100-fold lower than that of strong ligand. ($K_{d,low} = 100K_{d,high}$).

SI Dataset 2: The simulation data of LecA binding kinetics when the affinity of the weak ligand is 300-fold lower than that of strong ligand. ($K_{d,low} = 300K_{d,high}$).

SI Dataset 3: The simulation data of LecA binding kinetics when the affinity of the weak ligand is 1000-fold lower than that of strong ligand. ($K_{d,low} = 1000K_{d,high}$).

REFRENCES

1 Tao, A., Sinsermsuksakul, P. & Yang, P. Polyhedral Silver Nanocrystals with Distinct Scattering Signatures. *Angewandte Chemie International Edition* **45**, 4597-4601, doi:10.1002/anie.200601277 (2006).

2 Wu, H. J. *et al.* Membrane-protein binding measured with solution-phase plasmonic nanocube sensors. *Nature methods* **9**, 1189-1191, doi:10.1038/nmeth.2211 (2012).

3 Worstell, N. C., Krishnan, P., Weatherston, J. D. & Wu, H. J. Binding Cooperativity Matters: A GM1-Like Ganglioside-Cholera Toxin B Subunit Binding Study Using a Nanocube-Based Lipid Bilayer Array. *PLoS One* **11**, e0153265, doi:10.1371/journal.pone.0153265 (2016).

4 Gomez, E. W., Clack, N. G., Wu, H. J. & Groves, J. T. Like-charge interactions between colloidal particles are asymmetric with respect to sign. *Soft Matter* **5**, 1931-1936, doi:10.1039/B821510C (2009).

5 Shi, J. *et al.* GM1 clustering inhibits cholera toxin binding in supported phospholipid membranes. *J Am Chem Soc* **129**, 5954-5961, doi:10.1021/ja069375w (2007).

6 Krishnan, P. *et al.* Hetero-multivalent binding of cholera toxin subunit B with glycolipid mixtures. *Colloids and surfaces. B, Biointerfaces* **160**, 281-288, doi:10.1016/j.colsurfb.2017.09.035 (2017).

7 Duncan, G. A. & Bevan, M. A. Tunable aggregation by competing biomolecular interactions. *Langmuir* **30**, 15253-15260, doi:10.1021/la503772g (2014).

8 Wu, H. J. & Bevan, M. A. Direct measurement of single and ensemble average particle-surface potential energy profiles. *Langmuir* **21**, 1244-1254, doi:10.1021/la047892r (2005).

9 Wu, H. J., Pangburn, T. O., Beckham, R. E. & Bevan, M. A. Measurement and interpretation of particle-particle and particle-wall interactions in levitated colloidal ensembles. *Langmuir* **21**, 9879-9888, doi:10.1021/la050671g (2005).

10 Everett, W. N., Beltran-Villegas, D. J. & Bevan, M. A. Concentrated diffusing colloidal probes of Ca2+-dependent cadherin interactions. *Langmuir* **26**, 18976-18984, doi:10.1021/la1038443 (2010).

11 Bevan, M. A. & Prieve, D. C. Direct measurement of retarded van der Waals attraction. *Langmuir* **15**, 7925-7936, doi:Doi 10.1021/La981381l (1999).

12 Pailthorpe, B. A. & Russel, W. B. The retarded van der Waals interaction between spheres. *Journal of Colloid and Interface Science* **89**, 563-566, doi:10.1016/0021-9797(82)90208-9 (1982).

13 Lewis, B. A. & Engelman, D. M. Lipid bilayer thickness varies linearly with acyl chain length in fluid phosphatidylcholine vesicles. *J Mol Biol* **166**, 211-217, doi:10.1016/S0022-2836(83)80007-2 (1983).

14 Redhead, H. M., Davis, S. S. & Illum, L. Drug delivery in poly(lactide-co-glycolide) nanoparticles surface modified with poloxamer 407 and poloxamine 908: in vitro characterisation and in vivo evaluation. *Journal of controlled release : official journal of the Controlled Release Society* **70**, 353-363, doi:10.1016/S0168-3659(00)00367-9 (2001).

15 Wu, H. J., Everett, W. N., Anekal, S. G. & Bevan, M. A. Mapping patterned potential energy landscapes with diffusing colloidal probes. *Langmuir* **22**, 6826-6836, doi:10.1021/la060501j (2006).

16 Anekal, S. G. & Bevan, M. A. Self-diffusion in submonolayer colloidal fluids near a wall. *J Chem Phys* **125**, 34906, doi:10.1063/1.2211616 (2006).

17 Honig, E. P., Roebersen, G. J. & Wiersema, P. H. Effect of hydrodynamic interaction on the coagulation rate of hydrophobic colloids. *Journal of Colloid and Interface Science* **36**, 97-109, doi:10.1016/0021-9797(71)90245-1 (1971).

18 Hardt, S. L. Rates of diffusion controlled reactions in one, two and three dimensions. *Biophys Chem* **10**, 239-243, doi:10.1016/0301-4622(79)85012-7 (1979).

19 Nayhouse, M., Kwon, J. S. I., Christofides, P. D. & Orkoulas, G. Crystal shape modeling and control in protein crystal growth. *Chemical Engineering Science* **87**, 216-223, doi:10.1016/j.ces.2012.10.020 (2013).

20 Fallahi-Sichani, M. & Linderman, J. J. Lipid raft-mediated regulation of G-protein coupled receptor signaling by ligands which influence receptor dimerization: a computational study. *PLoS One* **4**, e6604, doi:10.1371/journal.pone.0006604 (2009).

21 Day, C. A. & Kenworthy, A. K. Mechanisms underlying the confined diffusion of cholera toxin B-subunit in intact cell membranes. *PLoS One* **7**, e34923, doi:10.1371/journal.pone.0034923 (2012).

22 Nayhouse, M. *et al.* Modeling and control of ibuprofen crystal growth and size distribution. *Chemical Engineering Science* **134**, 414-422, doi:10.1016/j.ces.2015.05.033 (2015).
23 Lindblom, G. & Oradd, G. Lipid lateral diffusion and membrane heterogeneity. *Biochim Biophys Acta* **1788**, 234-244, doi:10.1016/j.bbamem.2008.08.016 (2009).
24 Lauer, S., Goldstein, B., Nolan, R. L. & Nolan, J. P. Analysis of cholera toxin-ganglioside interactions by flow cytometry. *Biochemistry* **41**, 1742-1751, doi:10.1021/bi0112816 (2002).
25 Sengers, B. G. *et al.* Modeling bispecific monoclonal antibody interaction with two cell membrane targets indicates the importance of surface diffusion. *MAbs* **8**, 905-915, doi:10.1080/19420862.2016.1178437 (2016).
26 Breimer, M. E., Hansson, G. C., Karlsson, K. A., Larson, G. & Leffler, H. Glycosphingolipid composition of epithelial cells isolated along the villus axis of small intestine of a single human individual. *Glycobiology* **22**, 1721-1730, doi:10.1093/glycob/cws115 (2012).
27 Schnaar, R. L. & Kinoshita, T. in *Essentials of Glycobiology* (eds A. Varki *et al.*) (2015-2017).
28 Blanchard, B. *et al.* Structural basis of the preferential binding for globo-series glycosphingolipids displayed by Pseudomonas aeruginosa lectin I. *J Mol Biol* **383**, 837-853, doi:10.1016/j.jmb.2008.08.028 (2008).
29 Mahal, L. K., To generate specificity profiles for commercially available reagents for the community and to facilitate the comparison of glycan arrays on multiple platforms. Glycan array data in Consortium for Functional Glycomics, dataset number: primscreen_4787, primscreen_4788, primscreen_4789, primscreen_4790, 2011. Available from: www.functionalglycomics.com.
30 Lanne, B., Cîopraga, J., Bergström, J., Motas, C. & Karlsson, K.-A. Binding of the galactose-specificPseudomonas aeruginosa lectin, PA-I, to glycosphingolipids and other glycoconjugates. *Glycoconjugate Journal* **11**, 292-298, doi:10.1007/bf00731201 (1994).
